# An inducible circular RNA *circKcnt2* inhibits ILC3 activation to facilitate colitis resolution

**DOI:** 10.1038/s41467-020-17944-5

**Published:** 2020-08-14

**Authors:** Benyu Liu, Buqing Ye, Xiaoxiao Zhu, Liuliu Yang, Huimu Li, Nian Liu, Pingping Zhu, Tiankun Lu, Luyun He, Yong Tian, Zusen Fan

**Affiliations:** 1 Key Laboratory of Infection and Immunity of CAS, CAS Center for Excellence in Biomacromolecules, Institute of Biophysics, Chinese Academy of Sciences, 100101 Beijing, China; 2 Key Laboratory of RNA Biology, Institute of Biophysics, Chinese Academy of Sciences, 100101 Beijing, China; 3 University of Chinese Academy of Sciences, 100049 Beijing, China

**Keywords:** Gene regulation in immune cells, Inflammation, Innate lymphoid cells, Non-coding RNAs

## Abstract

Group 3 innate lymphoid cells (ILC3) are an important regulator for immunity, inflammation and tissue homeostasis in the intestine, but how ILC3 activation is regulated remains elusive. Here we identify a new circular RNA (circRNA) *circKcnt2* that is induced in ILC3s during intestinal inflammation. Deletion of *circKcnt2* causes gut ILC3 activation and severe colitis in mice. Mechanistically, *circKcnt2*, as a nuclear circRNA, recruits the nucleosome remodeling deacetylase (NuRD) complex onto *Batf* promoter to inhibit Batf expression; this in turn suppresses *Il17* expression and thereby ILC3 inactivation to promote innate colitis resolution. Furthermore, *Mbd3*^−/−^*Rag1*^−/−^ and *circKcnt2*^−/−^*Rag1*^−/−^ mice develop severe innate colitis following dextran sodium sulfate (DSS) treatments, while simultaneous deletion of *Batf* promotes colitis resolution. In summary, our data support a function of the circRNA *circKcnt2* in regulating ILC3 inactivation and resolution of innate colitis.

## Abstract (web summary)

Type 3 innate lymphoid cells (ILC3) are involved in maintaining gut immune homeostasis. Here the authors identify a circular RNA, *circKcnt2*, to be induced in ILC3s from inflamed gut, yet *circKcnt2* deletion aggravates mouse experimental colitis, thereby implicating *circKcnt2* as a potential feedback regulator of ILC3 activation and gut immunity.

## Introduction

Innate lymphoid cells (ILCs) mainly reside in mucosal surfaces and play a critical role in sustaining mucosal integrity and promoting lymphoid organogenesis^1,2^. According to their specific fate-decision transcription factors (TFs) and secreted cytokines, ILCs can be classified into three subgroups, including group 1 ILC (ILC1s), group 2 ILC (ILC2s), and group 3 ILC (ILC3s)^1,3^. Among them, ILC3s producing interleukin-22 (IL-22), IL-17, and interferon-γ (IFN-γ) are essential for maintenance of intestinal homeostasis^4,5^. Accumulating evidence indicates ILC3s are implicated in intestinal diseases, such as inflammatory bowel disease (IBD)^6^. Increased secretion of IL-22 by ILC3s facilitates resolution of microbial infection and tissue injury in the gut^5,7^. Nevertheless, excessive activation of ILC3s has been reported to induce colitis and cancer^6,8^. ILC3s in *Rag* (VDJ recombination activation gene)-deficient mice can cause innate colitis after anti-CD40 or dextran sodium sulfate (DSS) treatment^9,10^. In fact, ILC3 activation and inactivation in vivo are finely regulated to maintain gut homeostasis. For example, IL-23-induced STAT3 phosphorylation is a major pathway for ILC3 activation^11^. Treg cells can suppress ILC3-mediated colitis in an IL-10-independent fashion^12^. We previously showed that ILCregs inhibit ILC3 activation via secretion of IL-10 (ref. ^13^). However, whether and how noncoding RNA modulates ILC3 activity are unclear.

Circular RNAs (circRNAs), a class of noncoding RNAs, are characterized by a covalent bond linking the 3′ and 5′ ends formed by back-splicing^14^. CircRNAs were previously considered as byproducts of RNA splicing^15^. CircRNAs are generated by exons, introns, or exon–introns^16^. It has been reported that circRNAs extensively exist in various tissues and their expression profiles exhibit tissue and cell-type-specific patterns^17,18^. Moreover many circRNAs are highly conserved across species^19^. CircRNAs play critical roles in a variety of biological processes. For instance, ciRS-7 and Sry serve as miRNA sponges to exert their functions in brain^20^. Some exon-intronic circRNAs are located in the nucleus that are involved in the regulation of gene transcription by interacting with RNA polymerase II (ref. ^21^). CircRNAs are also implicated in the modulation of tumorigenesis^22^. We previously showed that a circRNA cia-cGAS associates with DNA sensor cGAS in the nuclei of hematopoietic stem cells (HSC) to block its synthase activity, maintaining HSC hemeostasis^18^. We recently demonstrated that circPan3 can promote the self-renewal of intestinal stem cells through IL-13 produced by niche ILC2s^18,23^.

Basic leucine zipper transcription factor ATF-like (Batf) belongs to the activator protein 1 (AP-1) family of TFs^24^. Accumulating evidence shows that Batf modulates adaptive immunity as a critical regulator. Batf participates in the regulation of differentiation of Th17, Th9, and follicular helper T (Tfh) cells^25^. Batf also modulates the class-switch recombination of B cells^26^. In addition, Batf deficiency reduces production of IFN-γ by CD8^+^ T cells^27^. Batf upregulation can suppress the effector function of exhausted CD8^+^ T cells^28^. Moreover, Batf is also involved in the regulation of regulatory T (T_reg_) cell development and maintanance^29,30^. However, the role of Batf in ILCs remains elusive.

Here we show that the circRNA *circKcnt2* (originating from *Knct2* gene transcript; gene symbol, mmu_circRNA_012594 or mmu_circ_0000084 in circBase) is highly induced in activated ILC3s during intestinal innate inflammation. *CircKcnt2* recruits the NuRD complex onto *Batf* promoter to suppress its expression, which inhibits ILC3 activation to promote innate colitis resolution. These results discover a previously unidentified contribution of circRNA to the regulation of colitis.

## Results

### *CircKcnt2* is highly induced in ILC3s during inflammation

**Fig. 1:**
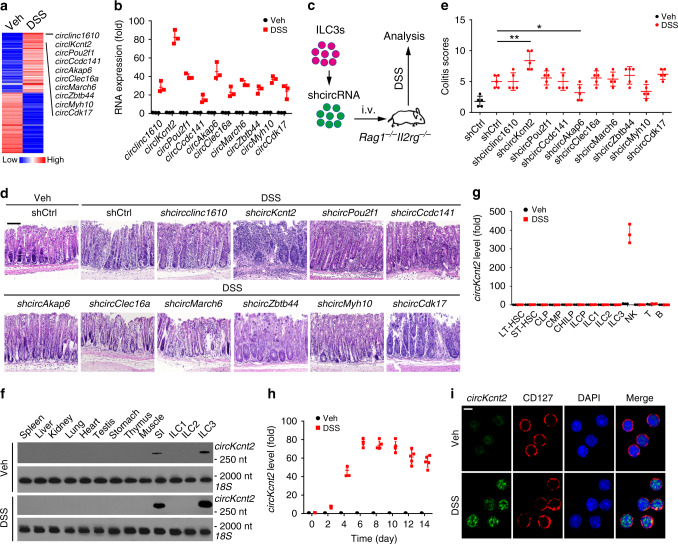
*CircKcnt2* is highly induced in ILC3s during intestinal inflammation. **a** Heatmap of differentially expressed circRNAs (100 most upregulated and 100 most downregulated) in small intestinal ILC3s (Lin^−^CD45^low^CD90^high^) from *Rag1*^−/−^ mice treated with or without DSS. ILC3s in intestines were isolated by FACS, followed by circRNA microarray analysis. Veh vehicle. **b** qRT-PCR analysis for top ten upregulated circRNAs in small intestinal ILC3s from DSS-treated *Rag1*^−/−^ mice. *n* = 3 independent samples. **c** ILC3s isolated from DSS-treated *Rag1*^−/−^ mice were infected with lentiviruses carrying shRNAs against circRNAs for 48 h. Then 3 × 10^4^ GFP^+^ ILC3s per mouse were transplanted into *Rag1*^−/−^*Il2rg*^−/−^ mice. Two weeks later, *Rag1*^−/−^*Il2rg*^−/−^ mice were treated with DSS, followed by analysis. **d** H&E staining of colons from transplanted *Rag1*^−/−^*Il2rg*^−/−^ mice as treated in **c**. Scale bar, 100 μm. **e** Colitis scores of indicated mice after DSS treatment as in **c** (*P = 0.0399; **P = 0.0031). *n* = 5 for each group. **f** Northern blotting analysis for *circKcnt2* expression in different tissues from *Rag1*^−/−^ mice treated with or without DSS. **g** qRT-PCR analysis for *circKcnt2* expression in indicated hematopoietic cells isolated from WT mice treated with or without DSS. *n* = 3 independent samples. **h** *CircKcnt2* expression in ILC3s from *Rag1*^−/−^ mice treated with 3% DSS was analyzed at different time points. *n* = 5 independent samples. **i** ILC3s were isolated from *Rag1*^−/−^ mice treated with 3% DSS and then *circKcnt2* expression was detected by fluorescence in situ hybridization (FISH). Biotin-labeled head-to-tail RNA probes were in green. Nuclei were counterstained with DAPI. Scale bar, 5 μm. *P < 0.05 and ***P < 0.001. Data were analyzed by an unpaired Student’s *t*-test and shown as means ± SD. Data are representative of at least three independent experiments. Source data are provided as a Source Data file.

To explore how circRNAs regulate ILC3 function in innate colitis, we isolated ILC3s from DSS-treated and control *Rag1*^−/−^ mice, followed by circRNA microarray analysis. With DSS treatment, many differentially expressed circRNAs appeared in *Rag1*^−/−^ ILC3s (Fig. 1a). We then chose top ten most upregulated circRNAs for validation. Their identities as circRNAs were further verified by PCR (Supplementary Fig. 1a) and RNase R digestion (Supplementary Fig. 1b). These ten circRNAs were really highly expressed in ILC3s isolated from DSS-treated *Rag1*^−/−^ mice through quantitative reverse transcriptase PCR (qRT-PCR) analysis (Fig. 1b). To further determine the functions of these ten circRNAs in ILC3s, we isolated ILC3s from DSS-treated *Rag1*^−/−^ mice and silenced them by shRNAs targeting head-to-tail regions, and then transplanted these cells into *Rag1*^−/−^*Il2rg*^−/−^ mice, followed by DSS treatment (Fig. 1c). Silencing efficiencies of these ten circRNAs with shRNAs were validated by qRT-PCR (Supplementary Fig. 1c). However, circRNA depletion did not affect the expression levels of their cognate linear transcripts (Supplementary Fig. 1d). Of these ten circRNAs, *circKcnt2* depletion caused more aggravated colon injury, lymphocyte infiltration (Fig. 1d), and more severe innate colitis (Fig. 1e). *CircPou2f1* or *circCcdc141* knockdown caused slight intestinal inflammation (Fig. 1d), but they did not cause statistical significant innate colitis compared to shCtrl treated mice (Fig. 1e). In addition, circAkap6 or circMyh10 depletion caused slight innate colitis (Fig. 1e). Here we focused on the role of *circKcnt2* in the regulation of innate colitis.

*CircKcnt2* is formed by back-splicing of *Kcnt2* transcripts from exon 4 to exon 8. We confirmed the back-splicing site through sequencing entire *circKcnt2* transcript (Supplementary Fig. 1e). Of note, *circKcnt2* was highly and specifically expressed in intestinal ILC3s through northern blot and qRT-PCR analyses (Fig. 1f, g). In addition, *circKcnt2* expression was gradually elevated in ILC3s during DSS treatment, reaching a peak level on day 6 (Fig. 1h). *CircKcnt2* was mainly distributed in the nuclei of ILC3s isolated from DSS-treated *Rag1*^−/−^ mice (Fig. 1i, Supplementary Fig. 1f). Finally, *circKcnt2* was highly conserved across species (Supplementary Fig. 1g). The back-splicing site of human *circKCNT2* was verified by DNA sequencing (Supplementary Fig. 1h). Collectively, *circKcnt2* is highly induced in ILC3s during innate colitis.

### *CircKcnt2* knockout induces ILC3 activation and colitis

To further determine the physiological role of *circKcnt2*, we next sought to generate *circKcnt2*-deficient mice. The complementary elements flanking circRNA sequences were essential for their formation^16^. We then screened out the complementary sequences in the introns flanking *circKcnt2* and analyzed their conservation across species (Supplementary Fig. 2a–c). Through minigene assay, we verified that the complementary sequences against *circKcnt2* were required for its formation (Supplementary Fig. 2d). We next generated *circKcnt2* knockout (*circKcnt2*^−/−^) mice through deleting the complementary intron sequences of genome with CRISPR/CAS9 technology (Supplementary Fig. 2e). *CircKcnt2* was successfully deleted in *circKcnt2*^−/−^ mice, while its maternal gene *Kcnt2* mRNA was not affected (Supplementary Fig. 2f–h). Furthermore, Kcnt2 expression was similar in *circKcnt2*^−/−^ mice versus WT mice (Supplementary Fig. 2h, i).

**Fig. 2:**
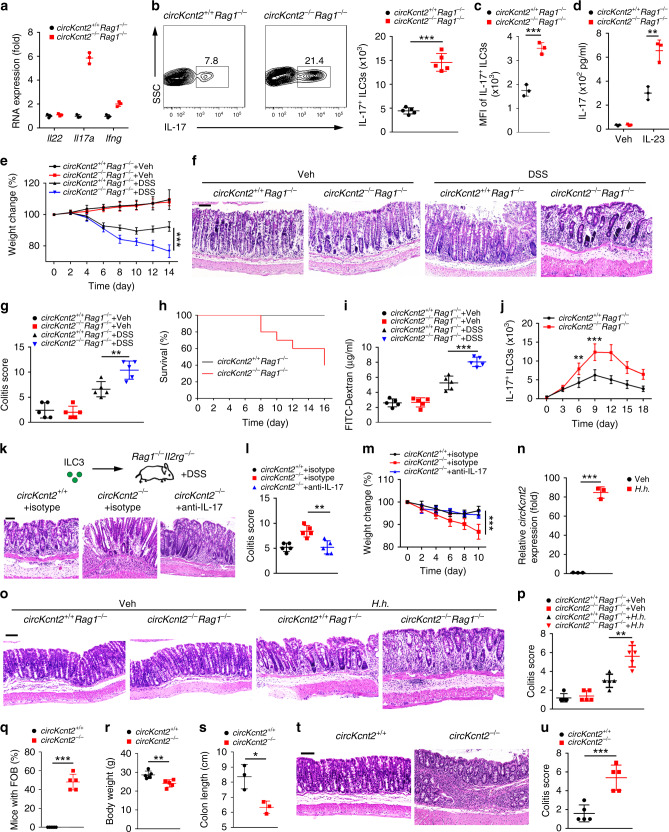
*CircKcnt2* knockout induces ILC3 activation and causes spontaneous colitis. **a** mRNA levels of indicated genes were tested by qRT-PCR in ILC3s from DSS-treated *circKcnt2*^+/+^*Rag1*^−/−^ and *circKcnt2*^−/−^*Rag1*^−/−^ mice. **b** Analysis of IL-17^+^ ILC3s (Lin^−^CD45^+^RORγt^+^ gated) in DSS-treated *circKcnt2*^+/+^*Rag1*^−/−^ and *circKcnt2*^−/−^*Rag1*^−/−^ mice (***P < 0.0001). Numbers of total and IL-17^+^ ILC3s were calculated in the right panels. *n* = 5 for each group. **c** Geometric mean fluorescence intensity (MFI) of IL-17^+^ ILC3s was shown (***P = 0.0008). *n* = 3 independent samples. **d** ILC3s were isolated from DSS-treated mice and incubated with or without IL-23 for 24 h (**P = 0.0041). Secreted IL-17 was detected by ELISA. Veh vehicle. *n* = 3 independent samples. **e** Body weight changes were calculated. *CircKcnt2*^+/+^*Rag1*^−/−^ and *circKcnt2*^−/−^*Rag1*^−/−^ mice were treated with 3% DSS for 7 days, followed by normal water for 7 days (***P = 0.0001). *n* = 5 for each group. **f** After DSS treatment, colon sections from *circKcnt2*^+/+^*Rag1*^−/−^ and *circKcnt2*^−/−^*Rag1*^−/−^ mice were analyzed by H&E staining. Scale bar, 100 μm. **g** Colitis scores of indicated mice were analyzed (**P = 0.0071). *n* = 5 for each group. **h** After DSS treatment, survival rates of *circKcnt2*^+/+^*Rag1*^−/−^ and *circKcnt2*^−/−^*Rag1*^−/−^ mice were monitored. *n* = 10 for each group. **i** *CircKcnt2*^+/+^*Rag1*^−/−^ and *circKcnt2*^−/−^*Rag1*^−/−^ mice treated with 3% DSS for 5 days were administrated with FITC-dextran by gavage. Translocation of FITC-dextran into mouse serum was detected (***P = 0.0005). *n* = 5 independent samples. **j** IL-17^+^ ILC3 numbers were calculated in *circKcnt2*^+/+^*Rag1*^−/−^ and *circKcnt2*^−/−^*Rag1*^−/−^ mice after DSS treatment (**P = 0.0035; ***P = 0.0008). *n* = 5 for each group. **k** H&E staining of colons from indicated mice treated with 3% DSS and administrated with anti-IL-17 or isotype IgG. Scale bar, 100 μm. *CircKcnt2*^+/+^ and *circKcnt2*^−/−^ ILC3s (3 × 10^4^ cells per mouse) were transplanted into *Rag1*^−/−^*Il2rg*^−/−^ mice. Two weeks later, mice were treated with DSS. **l** Colitis scores of indicated mice treated as in **k** were analyzed (**P = 0.0033). *n* = 5 for each group. **m** Body weight changes of mice in **k** were calculated (***P = 0.0005). *n* = 5 for each group. **n** *CircKcnt2* expression was tested by qRT-PCR in ILC3s from *H.h*.-infected *Il10*^−/−^ mice (***P < 0.0001). *n* = 3 independent samples. **o** After *H.h*. infection, colon sections from *circKcnt2*^+/+^*Rag1*^−/−^ and *circKcnt2*^−/−^*Rag1*^−/−^ mice were analyzed by H&E staining. Scale bar, 100 μm. **p** Colitis scores of indicated mice were analyzed. *CircKcnt2*^+/+^*Rag1*^−/−^ and *circKcnt2*^−/−^*Rag1*^−/−^ mice were infected with *H.h*. (**P = 0.0025). *n* = 5 independent samples. **q** Frequencies of fecal occult blood (FOB) in *circKcnt2*^+/+^ and *circKcnt2*^−/−^ mice were analyzed (***P < 0.0001). *n* = 5 independent samples. **r** Body weights of *circKcnt2*^+/+^ and *circKcnt2*^−/−^ mice were measured (**P = 0^.^0065). *n* = 5 independent samples. **s** Colon lengths of *circKcnt2*^+/+^ and *circKcnt2*^−/−^ mice were detected (*P = 0.0176^)^. *n* = 3 independent samples. **t** H&E staining of colon sections from *circKcnt2*^+/+^ and *circKcnt2*^−/−^ mice. Scale bar^,^ 100 μm. **u** Colitis scores of *circKcnt2*^+/+^ and *circKcnt2*^−/−^ mice (***P ^=^ 0.0007). *n* = 5 for each group. *P < 0.05, **P < 0.01 and ***P < 0.001. Data were analyzed by an unpaired Student’s *t*-test and shown as means ± SD. Data are representative of at least three independent experiments. Source data are provided as a Source Data file.

We firstly tested the effect of *circKcnt2* on ILC development. We found that *circKcnt2*^−/−^ mice exhibited comparable numbers of ILC progenitors, including CLPs, CHILPs, and ILCPs in bone marrow (BM) to *circKcnt2*^+/+^ littermate WT mice (Supplementary Fig. 3a–c). Moreover, the numbers of NKs, ILC1s, ILC2s, and ILC3s were also not affected by *circKcnt2* deletion (Supplementary Fig. 3d–h). We thus wanted to determine whether *circKcnt2* deletion affected ILC3 activation during colitis induction. We then generated *circKcnt2*^−/−^*Rag1*^−/−^ mice through crossing *circKcnt2*^−/−^ mice with *Rag1*^+/−^ mice, followed by DSS treatment. We found that ILC3s of *circKcnt2*^−/−^*Rag1*^−/−^ mice expressed much more amount of *Il17a* after DSS treatment compared to *circKcnt2*^+/+^*Rag1*^−/−^ mice (Fig. 2a). Furthermore, IL-17^+^ ILC3 numbers in DSS-treated *circKcnt2*^−/−^*Rag1*^−/−^ mice were much greater than those of *circKcnt2*^+/+^*Rag1*^−/−^ mice (Fig. 2b). Of note, IL-17^+^ ILC3s of *circKcnt2*^−/−^*Rag1*^−/−^ mice did not undergo cell death (Supplementary Fig. 3i). In addition, MFI of IL-17^+^ ILC3s from DSS-treated *circKcnt2*^−/−^*Rag1*^−/−^ mice was much higher than that of *circKcnt2*^+/+^*Rag1*^−/−^ mice (Fig. 2c), suggesting that *circKcnt2*^−/−^*Rag1*^−/−^ ILC3s endowed IL-17-producing potential. In fact, *circKcnt2*^−/−^*Rag1*^−/−^ ILC3s produced more substantial amounts of IL-17 than *circKcnt2*^+/+^*Rag1*^−/−^ ILC3s (Fig. 2d). We next overexpressed *circKcnt2* in *circKcnt2*^−/−^ ILC3s for rescue assay. As expected, *circKcnt2* was really restored in *circKcnt2*^−/−^ ILC3s (Supplementary Fig. 3j). Restoration of *circKcnt2* in *circKcnt2*^−/−^*Rag1*^−/−^ ILC3s could inhibit IL-17 generation (Supplementary Fig. 3k). These data indicate that *circKcnt2* suppresses IL-17 production in ILC3s.

It has been reported that IL-17 produced by ILC3s is an important regulator for innate colitis^8,13^. We next sought to explore whether *circKcnt2* participated in innate inflammation via modulating ILC3 activation. We noticed that *circKcnt2*^−/−^*Rag1*^−/−^ mice lost more body weight than *circKcnt2*^+/+^*Rag1*^−/−^ mice after DSS treatment (Fig. 2e). And *circKcnt2*^−/−^*Rag1*^−/−^ mice displayed more severe colon injury and more substantial lymphocytes infiltration than *circKcnt2*^+/+^*Rag1*^−/−^ mice (Fig. 2f). Furthermore, *circKcnt2*^−/−^*Rag1*^−/−^ mice exhibited much higher colitis scores and much lower survival rates (Fig. 2g, h). In addition, with adoptive transfer assay, engraftment of *circKcnt2*-deficient ILC3s with *circKcnt2* overexpression could attenuate innate colitis (Supplementary Fig. 3l, m). FITC-dextran by gavage is used to measure mouse intestinal damage^8^. We observed that *circKcnt2*^−/−^*Rag1*^−/−^ mice exhibited much higher concentrations of blood FITC-dextran (Fig. 2i), suggesting severe intestinal injury of *circKcnt2*^−/−^*Rag1*^−/−^ mice. Therefore, *circKcnt2* deletion promotes induction of mouse innate colitis.

In addition, IL-17^+^ ILC3 numbers were gradually increased in *circKcnt2*^−/−^*Rag1*^−/−^ mice with DSS treatment, reaching a peak number at day 9 in line with a peak level of innate inflammation (Fig. 2j). We next isolated *circKcnt2*^+/+^ and *circKcnt2*^−/−^ ILC3s and transplanted them into *Rag1*^−/−^*Il2rg*^−/−^ mice to test their roles in the regulation of innate inflammation. Two weeks later, recipient mice were administrated with anti-IL-17 antibody or isotype IgG, followed by DSS treatment. We noticed that recipient mice with *circKcnt2*^−/−^ ILC3 transplantation showed much more severe colon injury and lymphocytes infiltration (Fig. 2k), and consequently exhibited higher colitis scores and more severe body weight loss (Fig. 2l, m). However, anti-IL-17 antibody treatment attenuated innate colitis scores and body weight loss of *Rag1*^−/−^*Il2rg*^−/−^ mice (Fig. 2k–m). To exclude the effect of Kcnt2 on innate colitis induction, we established Kcnt2 KO mice via CRISPR/Cas9 technology (Supplementary Fig. 4a). We found that Kcnt2 deletion did not affect *circKcnt2* expression (Supplementary Fig. 4b). We next generated *Kcnt2*^−/−^*Rag1*^−/−^ mice through crossing *Kcnt2*^−/−^ mice with *Rag1*^+/−^ mice, followed by DSS treatment. We observed that *Kcnt2*^−/−^*Rag1*^−/−^ mice did not affect lymphocytes infiltration, colon injury, body weight loss and colitis scores compared with *Kcnt2*^+/+^*Rag1*^−/−^ mice (Supplementary Fig. 4c–e), suggesting Kcnt2 has no influence on innate colitis induction.

*Helicobacter hepaticus* (*H.h*.)-induced chronic colitis is IL-17 dependent^9^. *H.h*. infection can cause colitis in *Il10*^−/−^ mice^31^. We first determined whether circKcnt2 expression was also induced by *H.h*. infection. *Il10*^−/−^ mice were infected with *H.h*., followed by detection of *circKcnt2* expression in ILC3s. We noticed that *circKcnt2* was markedly upregulated in ILC3s from *H.h*. infected *Il10*^−/−^ mice (Fig. 2n). We then infected *circKcnt2*^+/+^*Rag1*^−/−^ and *circKcnt2*^−/−^*Rag1*^−/−^ mice with *H.h*. to confirm the role of *circKcnt2* in IL-17-induced chronic colitis. We found that *circKcnt2*^−/−^*Rag1*^−/−^ mice displayed more substantial infiltration of inflammatory cells and higher colitis scores after 8 week challenge than *circKcnt2*^+/+^*Rag1*^−/−^ mice (Fig. 2o, p). Intestinal colonization of *H.h*. was comparable between *circKcnt2*^+/+^*Rag1*^−/−^ and *circKcnt2*^−/−^*Rag1*^−/−^ mice (Supplementary Fig. 4f), suggesting that this colitis discrepancy was not due to increased intestinal colonization by *H.h*. More importantly, *circKcnt2*^−/−^ mice displayed rectal bleeding, lower body weight and shorter colon length at about 8 months of age (Fig. 2q–s), as well as more infiltration of inflammatory cells and higher colitis scores (Fig. 2t, u), indicating *circKcnt2*^−/−^ mice underwent spontaneous colitis. Collectively, *circKcnt2* knockout induces ILC3 activation and promotes innate intestinal colitis.

### *CircKcnt2* suppresses *Batf* transcription

**Fig. 3:**
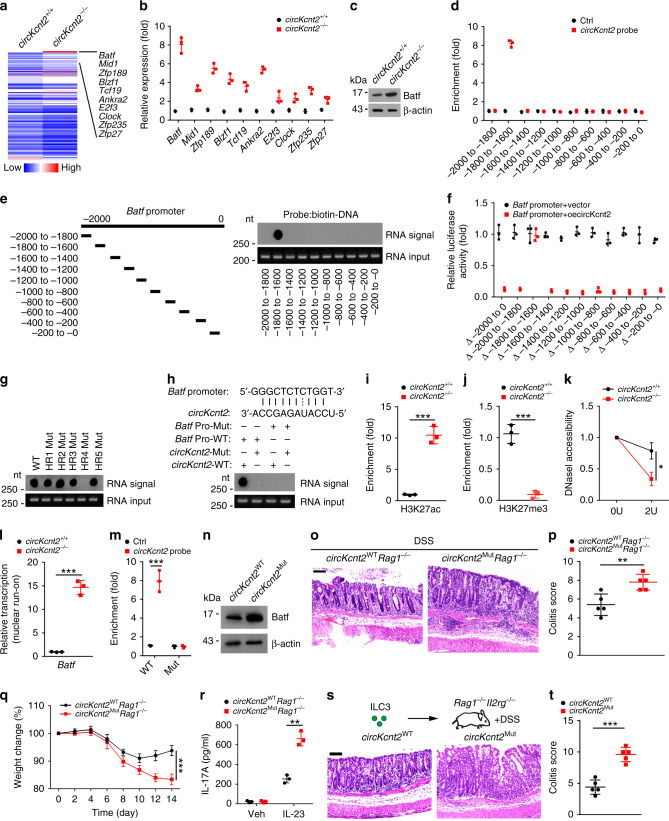
*CircKcnt2* suppresses *Batf* transcription. **a** Heatmap analysis according to differentially expressed TFs in *circKcnt2*^+/+^ and *circKcnt2*^−/−^ ILC3s isolated from DSS-treated mice. **b** ILC3s were isolated from *circKcnt2*^+/+^ and *circKcnt2*^−/−^ mice after DSS treatment, followed by RNA extraction and qRT-PCR analysis for top ten upregulated TFs. *n* = 3 independent samples. **c** Batf expression was tested by western blotting in *circKcnt2*^+/+^ and *circKcnt2*^−/−^ ILC3s isolated from DSS-treated mice. **d** ILC3s from DSS-treated mice were lyzed and subjected to CHIRP assay using biotin-labeled probes against *circKcnt2*. Precipitated DNA fragments were extracted and enrichment of *circKcnt2* on *Batf* promoter was analyzed by PCR. *n* = 3 independent samples. **e** *CircKcnt2* linearized RNA was immobilized on NC membranes, followed by probing with indicated biotin-labeled DNA probes. **f** Luciferase reporter assay using *Batf* truncated promoters with overexpression of *circKcnt2* or vector control. *n* = 3 independent samples. **g** *CircKcnt2* linearized RNAs were immobilized onto membrane, followed by probing with biotin-labeled single-strand DNA. HR hairpin region. **h** A base pairing between *Batf* promoter (−1800 to −1600) and *circKcnt2* HR4 was predicted. WT or mutant *circKcnt2* HR4 RNAs were immobilized onto membrane, followed by probing with WT or mutant DNA probes. **i**, **j** ChIP assay was performed using anti-H3K27ac or anti-H3K27me3 with *circKcnt2*^+/+^ and *circKcnt2*^−/−^ ILC3 cell lysates after DSS treatment (***P = 0.0003; ***P = 0.0006). *n* = 3 independent samples. **k** DNaseI accessibility assay was conducted using *circKcnt2*^+/+^ and *circKcnt2*^−/−^ ILC3s from DSS-treated mice (*P = 0.0102). *n* = 3 independent samples. **l** *CircKcnt2*^+/+^ and *circKcnt2*^−/−^ ILC3s were subjected to nuclear run-on assay, followed by detection of *Batf* transcription by RT-PCR analysis (***P = 0.0001). *n* = 3 independent samples. **m** *CircKcnt2* enrichment on *Batf* promoter was tested by CHIRP assay using *circKcnt2*^WT^ and *circKcnt2*^Mut^ ILC3 lysates after DSS treatment (***P = 0.0005). *n* = 3 independent samples. **n** Batf expression was measured using western blotting in *circKcnt2*^WT^ and *circKcnt2*^Mut^ ILC3s after DSS treatment. **o** H&E staining of colon tissues from *circKcnt2*^WT^*Rag1*^−/−^ and *circKcnt2*^Mut^*Rag1*^−/−^ mice treated with DSS. Scale bar, 100 μm. **p** Colitis scores of colon tissues in **o** (**P = 0.0053). *n* = 5 independent samples. **q** *CircKcnt2*^WT^*Rag1*^−/−^ and *circKcnt2*^Mut^*Rag1*^−/−^ mice were treated with DSS and weight changes were monitored (***P < 0.0001). *n* = 5 independent samples. **r** ILC3s were isolated from DSS-treated *circKcnt2*^WT^*Rag1*^−/−^ and *circKcnt2*^Mut^*Rag1*^−/−^ mice, followed by ELISA assay (**P = 0.0011). Veh vehicle. *n* = 3 independent samples. **s** ILC3s isolated from *circKcnt2*^WT^ and *circKcnt2*^Mut^ mice were transplanted into *Rag1*^−/−^*Il2rg*^−/−^ mice. Two weeks later, recipient mice were administrated with DSS. Colons were isolated and analyzed by H&E staining. Scale bar, 100 μm. **t** Colitis scores of indicated mice treated as in **s** were analyzed (***P < 0.0001). *n* = 5 for each group. *P < 0.05, **P < 0.01, and ***P < 0.001. Data were analyzed by an unpaired Student’s *t*-test and shown as means ± SD. Data are representative of at least three independent experiments. Source data are provided as a Source Data file.

To explore the molecular mechanism of *circKcnt2* in the regulation of ILC3 activity, we isolated *circKcnt2*^+/+^ and *circKcnt2*^−/−^ ILC3s from DSS-treated mice and performed transcriptome microarray analysis. We found that *circKcnt2* deletion caused upregulation of many TFs (Fig. 3a). Among top ten upregulated TFs, *Batf* was the most upregulated (Fig. 3a, b). Batf upregulation of *circKcnt2*^−/−^ ILC3s was also confirmed by western blot (Fig. 3c). Through chromatin isolation by RNA purification (CHIRP) assay, we observed that *circKcnt2* was enriched on the promoter region (−1800 to −1600) of *Batf* (Fig. 3d). We found that *circKcnt2* bound to the segment (−1800 to −1600) of *Batf* promoter via hybridization with *circKcnt2* linearized RNAs (Fig. 3e). This result was further validated by luciferase assay (Fig. 3f). These data suggest that *circKcnt2* binds *Batf* promoter to regulate its transcription.

CircRNA loops have critical roles in the RNA interactome^23^. We predicted the loop structure of *circKcnt2* with bioinformatics tool (Supplementary Fig. 4g). To determine which loop (HR1, HR2, HR3, HR4, and HR5) was required for the association between *circKcnt2* and *Batf* promoter, we constructed *circKcnt2* mutations to abrogate loop structures (Supplementary Table 1). We found that HR4 was required for their interaction (Fig. 3g). Through sequence alignment, we noticed that a pairing complementary bases existed between *Batf* promoter (−1800 to −1600) and HR4 of *circKcnt2* (Fig. 3h). We generated mutations of *Batf* promoter and *circKcnt2* transcript to abrogate their binding, followed by hybridization assay. We found that the binding base mutations really abolished their interactions (Fig. 3h). Through ChIP assay, we found that *circKcnt2* deletion promoted enrichment of H3K27ac on *Batf* promoter in ILC3s (Fig. 3i). By contrast, *circKcnt2* deletion decreased H3K27me3 enrichment on *Batf* promoter in ILC3s (Fig. 3j). To assess density of nucleosomes in *Batf* promoter, we performed DNaseI accessibility assay. As shown, *Batf* promoter in *circKcnt2*^−/−^ ILC3s was more susceptible to DNaseI digestion (Fig. 3k). Consistently, *circKcnt2* deficiency caused elevated transcription of *Batf* mRNA by nuclear run-on assay (Fig. 3l). These data indicate that *circKcnt2* interacts with *Batf* promoter to suppress its transcription.

To further confirm the in vivo role of their pairing bases, we generated *circKcnt2*-mutant (called *circKcnt2*^Mut^) mice through knocking in the mutation bases of pairing *circKcnt2* region (Supplementary Fig. 4h). We observed that *circKcnt2* mutation abrogated the enrichment of *circKcnt2* onto *Batf* promoter (Fig. 3m), and consequently enhanced *Batf* expression in ILC3s with DSS treatment (Fig. 3n). We then treated *circKcnt2*^WT^*Rag1*^−/−^ and *circKcnt2*^Mut^*Rag1*^−/−^ mice with DSS. We noticed that *circKcnt2*^Mut^*Rag1*^−/−^ mice displayed much more severe innate colitis compared to *circKcnt2*^WT^*Rag1*^−/−^ mice (Fig. 3o–q). In addition, *circKcnt2*^Mut^*Rag1*^−/−^ ILC3s produced more IL-17 in vitro (Fig. 3r). We also noticed that *circKcnt2*^Mut^*Rag1*^−/−^ mice did not affect ILC3 development (Supplementary Fig. 4i). *circKcnt2*^Mut^ ILC3s did not undergo apparent cell death after DSS treatment (Supplementary Fig. 4j). Finally, we isolated ILC3s from *circKcnt2*^Mut^ and *circKcnt2*^WT^ mice, and transplanted them into *Rag1*^−/−^*Il2rg*^−/−^ mice with DSS treatment. We found that mice engrafted with *circKcnt2*^Mut^ ILC3s exhibited severe innate colitis (Fig. 3s, t). Altogether, *circKcnt2* enriches onto *Batf* promoter to inhibit its expression leading to suppression of ILC3 activation.

### *CircKcnt2* associates with Mbd3

**Fig. 4:**
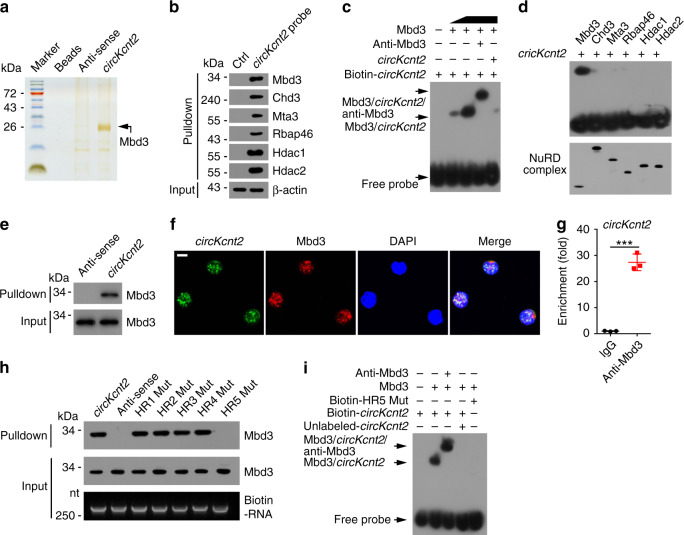
*CircKcnt2* associates with Mbd3. **a** LPL cells from DSS-treated WT mice were lysed and incubated with biotin-labeled and linearized *circKcnt2* transcripts, anti-sense, or Sepharose 4B beads control. Precipitants were resolved by SDS-PAGE, followed by silver staining. Indicated bands were identified via mass spectrometry. **b** LPL lysates were incubated with *circKcnt2* probes or controls, followed by western blotting using indicated antibodies. **c** Electrophoretic mobility shift assay (EMSA) using biotin-labeled *circKcnt2* and recombinant Mbd3 with or without anti-Mbd3. **d** EMSA assay using linearized *circKcnt2* RNAs and Flag tagged NuRD complex component proteins. **e** RNA pulldown assay was performed using linearized and biotin-labeled *circKcnt2* RNAs or anti-sense through LPL cell lysates. **f** *CircKcnt2* was co-localized with Mbd3 in ILC3s by immunofluorescence staining. Scale bar, 5 μm. **g** RNA immunoprecipitation (RIP) assay was conducted using anti-Mbd3 or IgG through LPL lysates from DSS-treated mice (***P = 0.0001). *n* = 3 independent samples. **h** RNA pulldown assay was performed using WT *circKcnt2* and its different mutations. **i** EMSA assay using WT *circKcnt2* or HR5 mutation. ***P < 0.001. Data were analyzed by an unpaired Student’s *t*-test and shown as means ± SD. Data are representative of at least three independent experiments. Source data are provided as a Source Data file.

To further reveal how *circKcnt2* suppressed *Batf* transcription in ILC3s, we conducted RNA pulldown assay using biotin-labeled linearized *circKcnt2* as bait, followed by silver staining and mass spectrometry. We found that *circKcnt2* bound to Mbd3 from gut lamina propria lymphocyte (LPL) lysates (Fig. 4a, Supplementary Fig. 5a). Mbd3 is a major component of the NuRD complex^32^. We noticed that *circKcnt2* could precipitate the main components of the NuRD complex in LPL lysates (Fig. 4b). The direct interaction between *circKcnt2* and Mbd3 was further validated by EMSA assay (Fig. 4c). Moreover, only Mbd3 of the NuRD complex components directly interacted with *circKcnt2* (Fig. 4d). The interaction between *circKcnt2* and Mbd3 in LPL lysates was further confirmed via RNA pulldown assay using biotin-labeled *circKcnt2* (Fig. 4e). Consistently, *circKcnt2* was co-localized with Mbd3 in ILC3s (Fig. 4f). In addition, *circKcnt2* was enriched by Mbd3 by RNA immunoprecipitation assay (Fig. 4g).

To further determine which loop of *circKcnt2* transcript was required for the interaction between *circKcnt2* and Mbd3, we performed RNA pulldown assay using biotin-labeled mutant *circKcnt2*. We observed that HR5 mutation abolished their association of *circKcnt2* with Mbd3 (Fig. 4h). This result was further validated by EMSA assay (Fig. 4i). Collectively, *circKcnt2* directly interacts with Mbd3.

### *CircKcnt2* recruits the NuRD complex onto *Batf* promoter

**Fig. 5:**
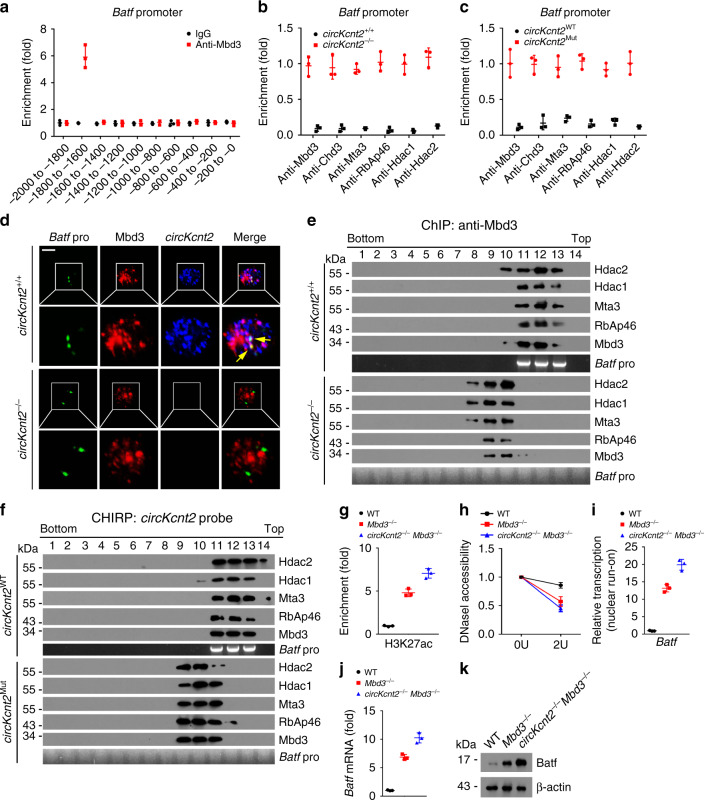
*CircKcnt2* recruits the NuRD complex onto *Batf* promoter to suppress its expression. **a** Analysis of Mbd3 enrichment on *Batf* promoter by ChIP assay. ILC3s was isolated from DSS-treated WT mice. *n* = 3 independent samples. **b** Enrichment analysis of NuRD complex component proteins on *Batf* promoter in *circKcnt2*^+/+^ and *circKcnt2*^−/−^ ILC3s after DSS treatment. *n* = 3 independent samples. **c** ChIP assay for enrichment of NuRD complex component proteins on *Batf* promoter in *circKcnt2*^WT^ and *circKcnt2*^Mut^ ILC3s from DSS-treated mice. *n* = 3 independent samples. **d** FISH assay for analysis of co-localization between Mbd3 and *Batf* promoter in *circKcnt2*^+/+^ and *circKcnt2*^−/−^ ILC3s from DSS-treated mice. Green, Batf promoter; red, Mbd3; blue, *circKcnt2*. Scale bar, 5 μm. **e** LPLs isolated from DSS-treated *circKcnt2*^+/+^ and *circKcnt2*^−/−^ mice were used for ChIP assay with anti-Mbd3. Eluents were subjected to sucrose gradient ultracentrifugation, followed by western blotting and PCR analysis. **f** LPLs were isolated from DSS-treated *circKcnt2*^WT^ and *circKcnt2*^Mut^ mice and used for CHIRP assay using biotin-labeled *circKcnt2* probes. **g** ChIP assay for enrichment analysis of H3K27ac on *Batf* promoter in *Mbd3*^−/−^, *circKcnt2*^−/−^*Mbd3*^−/−^, or WT control ILC3s. *n* = 3 independent samples. **h** DNaseI accessibility assay for *Batf* promoter accessibility in *Mbd3*^−/−^, *circKcnt2*^−/−^*Mbd3*^−/−^, or WT control ILC3s. *n* = 3 independent samples. **i** ILC3s from DSS-treated *Mbd3*^−/−^, *circKcnt2*^−/−^*Mbd3*^−/−^, or WT control mice were subjected to nuclear run-on assay, followed by RT-PCR analysis of *Batf*. *n* = 3 independent samples. **j**, **k** Analysis of Batf expression by qRT-PCR and western blotting in *Mbd3*^−/−^, *circKcnt2*^−/−^*Mbd3*^−/−^, or WT control ILC3s. *n* = 3 independent samples. Data were analyzed by an unpaired Student’s *t*-test and shown as means ± SD. Data are representative of at least three independent experiments. Source data are provided as a Source Data file.

We next sought to examine whether *circKcnt2* could recruit the NuRD complex onto *Batf* promoter to regulate its transcription. Through ChIP assay, we found that Mbd3 was enriched on the same region of *Batf* promoter as *circKcnt2* (Fig. 5a). However, *circKcnt2* deletion abrogated enrichment of the NuRD complex components on *Batf* promoter (Fig. 5b). Interestingly, the NuRD complex could not enrich on *Batf* promoter in *circKcnt2*^Mut^ ILC3s (Fig. 5c). Of note, Mbd3 co-localized with *Batf* promoter in WT ILC3s but not in *circKcnt2*^−/−^ ILC3s (Fig. 5d). Moreover, with cross-linking treatment, the NuRD complex was co-eluted with *Batf* promoter in *circKcnt2*^+/+^ LPL lysates, but not in *circKcnt2*^−/−^ LPL lysates (Fig. 5e). Through CHIRP assay, the NuRD complex and *Batf* promoter were co-eluted in *circKcnt2*^WT^ LPL lysates, but not in *circKcnt2*^Mut^ LPL lysates (Fig. 5f). These data indicate that *circKcnt2* recruits the NuRD complex onto *Batf* promoter in ILC3s.

To further test whether the NuRD complex regulated *Batf* transcription, we generated *Mbd3*^flox/flox^;*Rorc*-Cre (hereinafter referred to as *Mbd3*^−/−^) mice via crossing *Mbd3*^flox/flox^ mice with *Rorc*-Cre mice. Mbd3 was completely deleted in *Mbd3*^−/−^ mice (Supplementary Fig. 5b). We also generated *Mbd3*^−/−^*circKcnt2*^−/−^ mice through crossing *Mbd3*^−/−^ mice with *circKcnt2*^−/−^ mice. We found that *Batf* promoter enriched substantial H3K27ac in *Mbd3*^−/−^ and *Mbd3*^−/−^*circKcnt2*^−/−^ ILC3s (Fig. 5g). In addition, *Batf* promoter in *Mbd3*^−/−^ and *Mbd3*^−/−^*circKcnt2*^−/−^ ILC3s was more susceptible to DNaseI digestion compared to that of WT ILC3s (Fig. 5h). Consistently, *Batf* mRNA was more actively transcribed in *Mbd3*^−/−^ and *Mbd3*^−/−^*circKcnt2*^−/−^ ILC3s by nuclear run-on assay (Fig. 5i). Consequently, Batf was highly expressed in *Mbd3*^−/−^ and *Mbd3*^−/−^*circKcnt2*^−/−^ ILC3s (Fig. 5j, k). Taken together, *circKcnt2* recruits the NuRD complex to inhibit *Batf* transcription.

### *Batf* triggers ILC3 activation and colitis induction

**Fig. 6:**
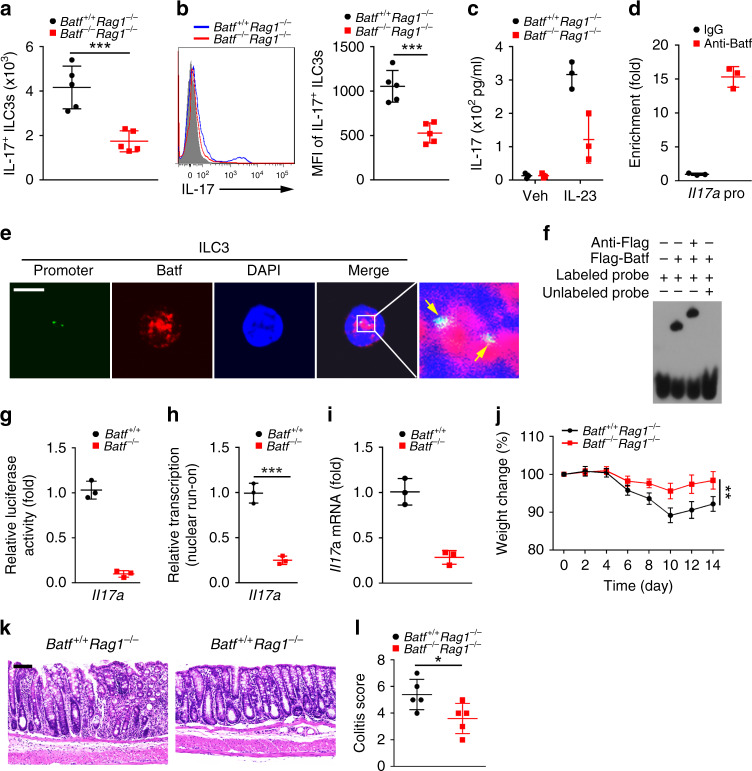
Batf triggers ILC3 activation and colitis induction. **a** IL-17^+^ ILC3 numbers were analyzed in *Batf*^+/+^*Rag1*^−/−^ or *Batf*^−/−^*Rag1*^−/−^ mice after DSS treatment (***P = 0.0009). *n* = 5 independent samples. **b** Expression of IL-17 was measured in *Batf*^+/+^*Rag1*^−/−^ or *Batf*^−/−^*Rag1*^−/−^ ILC3s from DSS-treated mice. Geometric MFI of IL-17^+^ ILC3s was presented in the right panel (***P = 0.0005). *n* = 5 independent samples. **c** ELISA assay using *Batf*^+/+^*Rag1*^−/−^ or *Batf*^−/−^*Rag1*^−/−^ ILC3s from DSS-treated mice. Veh vehicle. *n* = 3 independent samples. **d** Analysis of Batf enrichment on *Il17a* promoter in ILC3s by ChIP assay. *n* = 3 independent samples. **e** Co-localization between Batf and *Il17a* promoter was analyzed by FISH in ILC3s isolated from DSS-treated WT mice. Green, *Il17a* promoter; red, Batf; blue, nucleus. Scale bar, 5 μm. **f** The interaction between Batf and *Il17a* promoter was tested by EMSA assay. **g** Luciferase reporter assay was performed using *Il17a* reporter in *Batf*^+/+^ and *Batf*^−/−^ ILC3s. *n* = 3 independent samples. **h** *Batf*^+/+^ and *Batf*^−/−^ ILC3s were subjected to nuclear run-on assay, followed by RT-PCR analysis of *Il17a* (***P = 0.0004). *n* = 3 independent samples. **i** Relative expression of *Il17a* in ILC3s isolated from *Batf*^+/+^ and *Batf*^−/−^ mice treated with DSS. *n* = 3 independent samples. **j** Analysis of body weight changes of indicated mice after DSS treatment (***P = 0.0017). *n* = 5 for each group. **k** H&E staining of colons from indicated mice treated with DSS. Scale bar, 100 μm. **l** Colitis scores of indicated mice as in **k** (***P = 0.0372). *n* = 5 independent samples. *P < 0.05, **P < 0.01, and ***P < 0.001. Data were analyzed by an unpaired Student’s *t*-test and shown as means ± SD. Data are representative of at least three independent experiments. Source data are provided as a Source Data file.

To further examine the role of *Batf* in the regulation of ILC3s, we generated *Batf* knockout (KO) mice (Supplementary Fig. 5c). We then established *Batf*^−/−^*Rag1*^−/−^ mice via crossing *Batf*^−/−^ mice with *Rag1*^+/−^ mice, followed by DSS treatment for colitis induction. We observed that Batf deletion decreased numbers of IL-17^+^ ILC3s during intestinal inflammation (Fig. 6a). Moreover, *Batf*^−/−^*Rag1*^−/−^ ILC3s secreted less amounts of IL-17 (Fig. 6b, c). It has been reported that Batf can induce *Il17a* transcription in T cells^33^. We next sought to test whether Batf could initiate *Il17a* transcription in ILC3s. We found that Batf enriched on *Il17a* promoter in ILC3s (Fig. 6d). We noticed that Batf and *Il17a* promoter co-localized in ILC3s by fluorescence in situ hybridization assay (Fig. 6e). The direct interaction between Batf and *Il17a* promoter in ILC3s was further validated by EMSA (Fig. 6f). In addition, Batf promoted *Il17a* transcription in WT ILC3s via luciferase reporter assay (Fig. 6g). Of note, Batf deletion dramatically suppressed *Il17a* transcription (Fig. 6h, i). More importantly, *Batf*^−/−^*Rag1*^−/−^ mice exhibited mild intestinal inflammation and slight injury (Fig. 6j–l). Altogether, Batf initiates *Il17a* expression to induce ILC3 activation leading to colitis induction.

### *Batf* deletion promotes colitis resolution

**Fig. 7:**
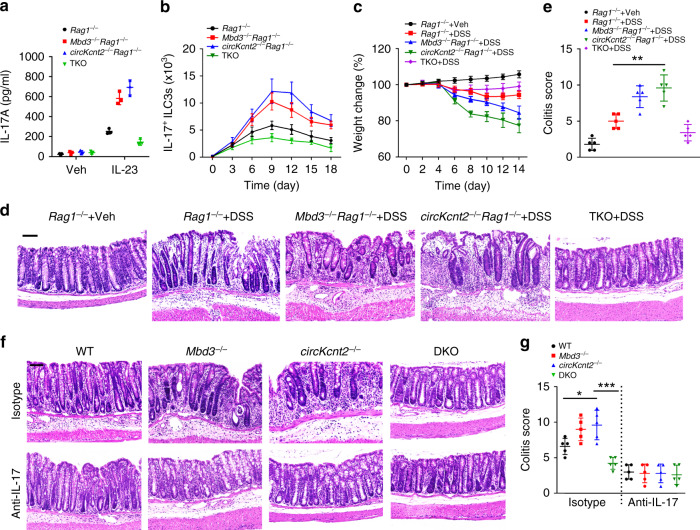
Mbd3 deletion causes severe innate colitis while Batf deletion promotes colitis resolution. **a** ILC3s isolated from indicated mice were used for ELISA assay to analyze IL-17 production. TKO, *circKcnt2*^−/−^*Batf*^−/−^*Rag1*^−/−^ mice. *n* = 3 independent samples. **b** Cell numbers of IL-17^+^ ILC3s were analyzed in indicated mice after DSS treatment. *n* = 5 for each group. **c** Analysis of body weight changes of indicated mice after DSS treatment. *n* = 5 for each group. **d** H&E staining of colons from indicated mice treated with DSS. Scale bar, 100 μm. **e** Colitis scores of indicated mice as in **d** (**P = 0.0011). **f** ILC3s (3 × 10^4^ cells per mouse) isolated from *Mbd3*^−/−^, *circKcnt2*^−/−^, *circKcnt2*^−/−^*Batf*^−/−^ (DKO), or WT control mice were transplanted into *Rag1*^−/−^*Il2rg*^−/−^ mice. Two weeks later, mice were administrated with DSS and anti-IL-17 or isotype IgG. Colons were isolated and analyzed by H&E staining. Scale bar, 100 μm. **g** Colitis scores of indicated mice as in **f** (*P = 0.0219; ***P = 0.0006). *n* = 5 independent samples. *P < 0.05 and **P < 0.01. Data are shown as means ± SD. Data were analyzed by an unpaired Student’s *t*-test and representative of at least three independent experiments. Source data are provided as a Source Data file.

To further determine the physiological role of Mbd3 in the modulation of ILC3s and intestinal innate colitis, we generated *Mbd3*^−/−^*Rag1*^−/−^ mice. We observed that *Mbd3*^−/−^*Rag1*^−/−^ ILC3s produced more substantial amounts of IL-17 after DSS challenge, similar to *circKcnt2*^−/−^*Rag1*^−/−^ ILC3s (Fig. 7a). Moreover, numbers of IL-17^+^ ILC3s were gradually increased in *Mbd3*^−/−^*Rag1*^−/−^ and *circKcnt2*^−/−^*Rag1*^−/−^ mice during intestinal inflammation (Fig. 7b). We also established *circKcnt2*^−/−^*Batf*^−/−^*Rag1*^−/−^ (TKO) mice. We noticed that TKO ILC3s produced much less amounts of IL-17 and TKO mice had lower numbers of IL-17^+^ ILC3s with DSS treatment than *Mbd3*^−/−^*Rag1*^−/−^ or *circKcnt2*^−/−^*Rag1*^−/−^ control mice (Fig. 7a, b). Consistently, *Mbd3*^−/−^*Rag1*^−/−^ and *circKcnt2*^−/−^*Rag1*^−/−^ mice displayed more severe lymphocyte infiltration and intestinal injury, whereas TKO mice exhibited opposite phenotypes (Fig. 7c–e). These data suggest that Mbd3 suppresses ILC3 activation that is opposite to Batf function.

We then transplanted *Mbd3*^−/−^, *circKcnt2*^−/−^, *circKcnt2*^−/−^*Batf*^−/−^ (hereinafter referred to as DKO) or WT ILC3s into *Rag1*^−/−^*Il2rg*^−/−^ mice, followed by DSS treatment with or without administration of anti-IL-17 antibody. We observed that adoptive transfer of *Mbd3*^−/−^ and *circKcnt2*^−/−^ ILC3s caused more severe innate colitis compared to WT ILC3s (Fig. 7f, g). By contrast, engraftment of DKO ILC3s exhibited slighter innate colitis than transfer of *Mbd3*^−/−^ or *circKcnt2*^−/−^ ILC3s alone (Fig. 7g, g). Of note, with anti-IL-17 antibody treatment, adoptive transfer of *Mbd3*^−/−^ and *circKcnt2*^−/−^ ILC3s induced attenuated innate colitis (Fig. 7f, g), indicating that IL-17 induced by ILC3s plays a critical role in *circKcnt2*-mediated inflammatory inhibition. Taken together, *circKcnt2* recruits the NuRD complex onto *Batf* promoter to suppress its expression, which inhibits ILC3 activation to facilitate innate colitis resolution.

## Discussion

ILC3s are an important regulator for immunity, inflammation, and tissue homeostasis in the intestine^34^. Dysregulation of ILC3 activation may lead to intestinal diseases, such as IBD^12^. However, how ILC3 activation is regulated remains elusive. In this study, we showed that *circKcnt2* is highly induced in activated ILC3s during intestinal innate colitis. Deletion of *circKcnt2* causes ILC3 activation and severe innate colitis. Mechanistically, *circKcnt2* as a nuclear circRNA recruits the NuRD complex onto *Batf* promoter to inhibit its expression, which suppresses *Il17* expression for ILC3 inactivation to promote innate colitis resolution (Supplementary Fig. 5d). Importantly, with DSS treatment, *Mbd3*^−/−^*Rag1*^−/−^ and *circKcnt2*^−/−^*Rag1*^−/−^ mice display severe innate colitis, whereas *Batf* deletion promotes colitis resolution. Therefore, we reveal a circRNA *circKcnt2* in regulating ILC3 inactivation and innate colitis resolution.

ILC3s are activated at the early stage of innate immune response that are important for pathogen elimination or tissue repair^1^. Deficiency or dysfunction of ILC3s leads to apparent pathologic disorders^35^. Similar to other immune responses, ILC3 activation also requires to be balanced by stringent regulations. Excessive activation of ILC3s may cause tissue damage and induce inflammatory disorders, such as IBD and tumorigenesis^9,36^. Several extrinsic factors are reported to be involved in the regulation of ILC3 activation. For example, IL-23 and IL-1β directly activates ILC3s to produce IL-22, which is essential for intestinal barrier integrity and mucosal healing^37^. IL-22 deficiency of ILC3s causes severe inflammation during *Citrobacter rodentium* infection or DSS-induced acute colitis^6^. TL1A derived from CX3CR1^+^ mononuclear phagocytes synergizes with IL-23 to enhance IL-22 secretion in ILC3s^6^. On the other hand, regulatory T cells indirectly restrain ILC3 function to alleviate colitis via inhibiting production of IL-23 and IL-1β by CX3CR1^+^ macrophages^12^. We previously demonstrated that ILCregs directly produce IL-10 to suppress ILC3 activation to facilitate resolution of intestinal inflammation^13^. As for intrinsic regulation, RANKL is reported to suppress ILC3 activity in the intestine via RANK–RANKL interactions between ILC3s^5^. However, how other intrinsic factors regulate ILC3 activity still remains unclear. Herein we identified an inducible circRNA *circKcnt2* in ILC3s during intestinal inflammation, which suppresses ILC3 activation to promote resolution of intestinal colitis. Our findings reveal that circRNAs played a crucial role in the regulation of ILC3 activity as intrinsic factors.

CircRNAs are a new class of noncoding RNA that are formed via covalent linkage of single RNA molecules by back-splicing^14,38^. CircRNAs are resistant to RNA exonucleases and possesses a longer half-life than other RNAs. Accumulating evidence indicates that circRNAs participate in the regulation of various biological processes via several mechanisms^39,40^. Some circRNAs specifically bind miRNAs as miRNA sponges to regulate gene expression^19,20^. In addition, some circRNAs exist in the nucleus and could regulate gene transcription^21,41^. We recently showed that circRNA cia-cGAS resides in the nuclei of HSCs to block the enzymatic activity of cGAS, which protects dormant HSCs from cGAS-mediated exhaustion^18^. We also identified circRNA circPan3 is highly expressed in intestinal stem cells (ISCs), which binds *Il13ra1* mRNA to increase its stability leading to the expression of IL-13Rα1 in ISCs for IL-13-IL-13R-mediated self-renewal maintenance of ISCs^23^. In this study, we showed that *circKcnt2* is upregulated in the nucleus of ILC3s over intestinal inflammation. *CircKcnt2* recruits the NuRD complex onto *Batf* promoter to suppress its expression, which inhibits ILC3 activation to facilitate resolution of innate colitis. Of note, Kcnt2, the parental gene of *circKcnt2*, is a potassium channel that specifically expresses in pain-sensing dorsal root ganglia neurons to reduce neuronal excitability^42^. We noticed that *circKcnt2* deletion does not affect Kcnt2 expression and Kcnt2 knockout has no effect on colitis induction, suggesting that *circKcnt2* exerts an independent role versus its parental gene.

ILC functions are finely regulated by both genetic and epigenetic aspects^43^. Chromatin remodeling is a prerequisite for eukaryotic gene transcription, depending on ATP-dependent remodeling complexes (also known as remodelers)^44^. These remodelers modulate chromatin structures via multiple means, including histone modifications, DNA methylations, and incorporation of histone variants. We previously showed that the Pcid2 controls lymphoid lineage development via modulating the activity of SRCAP remodeling complex^45^. We also demonstrated that the NURF remodeling complex is involved in the regulation of ILC maintenance^4^. The NuRD (nucleosome remodeling and deacetylase) complex, as a transcriptional repressor, forms repressive chromatin structure via the nucleosome remodeling (Mi-2α/β subunits) and histone deacetylase (HDAC1/2 subunits) activities^46,47^. Mbd3 deletion causes a defect of embryonic stem (ES) cell differentiation^32,48^. We previously found that Sox2 recruits the NuRD complex to inhibit mTOR expression, which drives autophagy induced cellular reprogramming^49^. Herein we showed that *circKcnt2* directly interacts with Mbd3 in ILC3s that recruits the NuRD complex onto Batf promoter, leading to inhibition of *Batf* transcription. Furthermore, Mbd3 deficiency causes severe colitis after DSS treatment. Our findings reveal that circRNAs-mediated chromatin remodeling by the NuRD complex plays a critical role in the regulation of ILC3 function.

Batf, a member of BATF family, is a basic leucine zipper TF that regulates gene transcription via associating with Jun proteins^25^. The functional investigations of Batf were previously mainly focused on T cells. Batf is involved in the development and differentiation of several types of T cells, such as Th2 and Th17 cells^26,33^. In addition, Batf is also implicated in the regulation of T cell functions. For instance, Batf enhances development of colitis-associated colon cancer relying on Th17 cells^50^. Batf also regulates intestinal graft-versus-host disease via IL-7R^hi^GM-CSF^+^ T cells^51^. Moreover, Batf deficiency causes reduced numbers of effector T and regulatory T cells in the intestine^52^. Here, we found that Batf promotes IL-17 expression of ILC3s to initiate ILC3 activation. *Batf* deletion suppresses ILC3 activation and attenuates innate colitis. In sum, *circKcnt2* recruits the NuRD complex onto *Batf* promoter in ILC3s to repress *Batf* transcription, which suppresses ILC3 activation and facilitate resolution of innate colitis.

## Methods

### Antibodies and reagents

Anti-H3K27ac (Cat# 8173), anti-Batf (Cat# 8638), anti-H3K27me3 (Cat# 9733), anti-Mbd3 (Cat# 99169), anti-Chd3 (Cat# 4241), anti-Rbap46 (Cat# 6882), anti-Hdac1 (Cat# 34589), anti-Hdac2 (Cat# 57156), anti-EEA1 (Cat# 3288), anti-H3 (Cat# 4499), and anti-β-actin (Cat# 3700) were from Cell Signaling Technology (Danvers, USA). Anti-Kcnt2 (Cat# bs-12177R) was from Bioss Antibodies (Beijing, China). Anti-CD127 (A7R34), anti-c-Kit (2B8), anti-CD3 (17A2), anti-CD4 (GK1.5), anti-CD19 (1D3), anti-NK1.1 (PK136), anti-CD150 (mShad150), anti-CD34 (RAM34), anti-CD45 (30-F11), anti-CD90 (HIS51), anti-Sca-1 (D7), anti-CD25 (PC61.5), anti-Flt3 (A2F10), anti-α_4_β_7_ (DATK32), anti-RORγt (AFKJS-9), anti-NKp46 (29A1.4), anti-Gata3 (TWAJ), anti-KLRG1 (2F1), anti-PLZF (Mags.21F7), Lineage cocktail (88-7772-72), anti-CD48 (HM48-1), Anti-IL-17 (eBio17B7), and anti-CD16/32 (93) were purchased from eBiosciences (San Diego, USA). Anti-BrdU (600-401-C29) was purchased from ThermoFisher. Paraformaldehyde (PFA) and 4′,6-diamidino-2-phenylindole (DAPI) were from Sigma-Aldrich. The IL-17 ELISA kit was purchased from eBiosciences.

### Generation of knockout mice by CRISPR/Cas9 technology

For generation of *circKcnt2*^−/−^, *Batf*^−/−^, *circKcnt2*^Mut^, and *Kcnt2*^−/−^ mice, CRISPR-mediated single-stranded oligodecxynucleotides donors were synthesized as previous described^53^. About 250 zygotes from C57BL/6 mice were injected with sgRNAs and subsequently transferred to the uterus of pseudo-pregnant ICR females from which viable founder mice were obtained. Genomic DNA mutation was identified by PCR screening and DNA sequencing, followed by western blotting or northern blotting. sgRNA sequences are listed in Supplementary Table 2. *Mbd3*^flox/flox^ mice were a gift from Dr. Brian Hendrich (University of Cambridge, UK). *Rorc*-Cre (022791), *PLZF*^GFPcre^ (024529), *Rorc*(γt)^+/GFP^ (007572), and *Id2*^+/GFP^ (016224) mice were purchased from the Jackson Laboratory. *Il10*^−/−^ (J002251) mice were purchased from Model Animal Research Center of Nanjing University, China. *Mbd3*^*f*lox/flox^*Rorc-*Cre mice were obtained by crossing *Mbd3*^flox/flox^ mice with *Rorc-*Cre mice. *Rag1*^−/−^*Il2rg*^−/−^ mice were generated by crossing *Rag1*^+/−^ mice (from Model Animal Research Center of Nanjing University, China; J002216) with *Il2rg*^+/−^ mice. All the mouse strains were of C57BL/6 background and were maintained under specific pathogen-free conditions with approval by the Institutional Committee of Institute of Biophysics, Chinese Academy of Sciences. The study is compliant with all relevant ethical regulations regarding animal research.

### Intestinal lymphocyte separation

Intestinal lymphocytes were obtained as previously reported^4^. Briefly, the Peyer’s patches were removed from the small intestine. Then the intestine was cut open longitudinally, followed by wash using phosphate buffer saline (PBS) five times. Afterwards, the intestine was cut into pieces and washed using solution I buffer (10 mM HEPES and 5 mM EDTA in HBSS) five times, followed by digestion using solution II buffer (DNaseI, 5% FBS, 0.5 mg/ml collagenase II and collagenase III) three times. Finally, LPLs were sifted through 70 μm strainers and utilized for experiments.

### Flow cytometry

BM cells were flushed out from femurs in PBS (containing 5% FBS) buffer and sifted through 70 μm cell strainers. LPL cells were isolated as described above. For flow cytometric analysis, LT-HSC (Lin^−^Sca-1^+^c-Kit^+^CD150^+^CD48^−^), ST-HSC (Lin^−^Sca-1^+^c-Kit^+^CD150^−^CD48^−^), CMP (Lin^−^Sca-1^−^c-Kit^+^CD34^+^CD16/32^−^), CLP (Lin^−^CD127^+^Sca-1^low^c-Kit^low^Flt3^+^), CHILP (Lin^−^CD127^+^Flt3^−^CD25^−^Id2^GFP^α_4_β_7_^+^), ILCP (Lin^−^Flt3^−^CD127^+^c-Kit^+^α_4_β_7_^+^PLZF^+^), ILC1 (CD3^−^CD19^−^CD127^+^NK1.1^+^NKp46^+^), ILC2 (Lin^−^CD127^+^CD90^+^KLRG1^+^Gata3^+^), ILC3 (Lin^−^RORγt^+^CD45^low^CD127^+^ or Lin^−^CD90^high^CD45^low^), NK (CD3^−^CD19^−^CD127^−^NK1.1^+^), CD19^+^ B, and CD3^+^ T populations were sorted with a FACSAria III instrument (BD Biosciences). *PLZF*^GFPcre^ mice were used for ILCPs isolation and *Id2*^+/GFP^ mice were used for CHILPs isolation by FACS.

### Immunofluorescence staining

ILC3s (Lin^−^CD90^high^CD45^low^) isolated by FACS were fixed with 4% PFA (Sigma-Aldrich) for 20 min at room temperature, perforated with PBS containing 1% TritonX-100 for 20 min, blocked with 5% donkey and 5% rat serum for 1 h at room temperature, incubated with appropriate primary antibodies at 4 °C overnight, and then incubated with fluorescence-conjugated secondary antibodies. DAPI was used for nucleus staining. Cells were visualized with Olympus FV1200 laser scanning confocal microscopy (Olympus, Japan).

### DNA fluorescence in situ hybridization

ILC3s (Lin^−^CD90^high^CD45^low^) were isolated and fixed with 4% PFA containing 10% acetic acid for 20 min at room temperature. Then cells were treated with 70% ethanol at −20 °C and incubated in buffer containing 100 mM Tris-HCl (pH 7.5), 150 mM NaCl, followed by cytoplasm digestion in 0.01% pepsin/0.01 N HCl for 3 min at 37 °C. Cells were further fixed with 3.7% PFA and replaced with ethanol to a final concentration of 100%. Afterwards, cells were air dried and washed using 2× SSC and blocked using buffer containing 100 mM Tris-HCl (pH 7.5), 150 mM NaCl, 0.05% Tween 20, 3% BSA for 20 min. Then, cells were denatured in 70% formamide/2× SSC and incubated with fluorescence-labeled DNA probes overnight. After staining with DAPI for nucleus, cells were visualized by Olympus FV1200 laser scanning confocal microscopy (Olympus, Japan).

### Real-time quantitative PCR

RNAs were extracted using a RNA Miniprep Kit (Tiangen, Beijing, China) according to the manufacturer’s protocol as previously described^54^. Then cDNA was synthesized with M-MLV reverse transcriptase (Promega, Madison, USA). Gene expression was analyzed on an ABI 7300 qPCR system using specific primer pairs listed in Supplementary Table 3. Relative expression levels were calculated and normalized to endogenous *18S* or *Gapdh*.

### Chromatin immunoprecipitation assay

ILC3s were cross-linked with 1% formaldehyde at 37 °C for 10 min. Then cells were washed twice with PBS, lysed with SDS lysis buffer (1% SDS, 10 mM EDTA, 50 mM Tris), and sonicated to make 200–500 bp DNA fragments. Lysates were pre-cleared with Protein A Agarose/Salmon Sperm DNA (50% Slurry) and then incubated with 4 μg antibody overnight at 4 °C. Then Protein A Agarose/Salmon Sperm DNA (50% Slurry) beads were added and incubated for 4 h. After washing, DNA was eluted from beads and purified. DNA fragments were analyzed using primer pairs listed in Supplementary Table 4. For ChIP immunoblotting assay, LPL cells were cross-linked with 1% formaldehyde and lysed with SDS lysis buffer and sonicated to 200–500 bp. Lysates were pre-cleared with protein A agarose/salmon sperm DNA (50% slurry). Then, 4 μg antibody was incubated with treated lysates for ChIP assays, followed by size fractionation with sucrose gradient ultracentrifugation. Five hundred microliters eluate was put onto 30 ml 5–30% (V/V) sucrose gradient followed by ultracentrifugation at 55,000*g* with an S Beckman SW28 rotor. Eluent gradients were concentrated and reversely cross-linked. Proteins were examined by western blotting. DNA was extracted and examined by PCR assays.

### DSS-induced colitis

For DSS-induced colitis, indicated mice (about 8 weeks) were administered 3% DSS in drinking water for 7 days, followed by 7 days of normal water.

### *Helicobacter hepaticus* infection

*Helicobacter hepaticus*, Hh NCI-Frederick isolate 1A (strain 51449; American Type Culture Collection), was used for colitis induction as previously reported^9^. In brief, *H.h*. was grown on blood agar plates containing trimethoprim, vancomycin, and polymyxin B (all from Oxoid) under microaerophilic conditions. For *H.h*. infection, indicated mice were infected with 1 × 10^8^ colony-forming unit *H.h*. by oral gavage for three times on alternate days. Colitis was analyzed 8 weeks later after the first *H. hepaticus* inoculation. For quantitation of *H.h*., DNA was purified from cecal contents taken from *H. hepaticus*-infected mice using the DNA Stool kit (Qiagen). *H.h*. DNA was determined using a Q-PCR method as previously described^55^.

### Colitis scores and histologic analysis

Colons were cut open longitudinally and fixed in 4% PFA followed by paraffin sectioning and H&E staining. The colitis score was calculated according to weight loss, appearance of the stool, intestinal bleeding, and histology as reported before^56^.

### Adoptive transfer assay

3 × 10^4^ ILC3s were adoptively transferred into *Rag1*^−/−^*Il2rg*^−/−^ mice. Two weeks later, transferred mice were treated with 3% DSS and colitis was then analyzed as described above.

### Lentivirus preparation and infection

RNA interference was performed as previously described^18^. And target sequences are listed in the Supplementary Table 5. For *circKcnt2* overexpression, genomic exon regions of *circKcnt2* were constructed into split GFP site on pSIN-EF2-GFP vector flanked with the upstream and downstream complementary elements. Lentiviral vector (pSIN-EF2-GFP) was co-transfected with packaging plasmids pVSVg and psPAX2 into HEK293T (ATCC® CRL-11268™) cells for 48 h, followed by culture medium collection and ultracentrifugation at 25,000*g* for 1.5 h. Pellets were resuspended in IMDM medium and viral titers were determined by infecting HEK293T cells with diluted viruses. Cells were incubated with lentiviruses (MOI = 10) and centrifuged at 500*g* for 2 h in the presence of 8 μg/ml polybrene. Cells were cultured for 24 h to allow GFP expression, followed by sorting of GFP-positive cells through a flow cytometer. HEK293T cells were from ATCC (CRL-11268) and tested negative for mycoplasma contamination.

### Immunoprecipitation assay

LPL cells were lysed and supernatants were incubated with biotin-labeled *circKcnt2* or antisence. Then precipitated components were separated with SDS-PAGE and silver staining. Differential bands enriched by *circKcnt2* were analyzed by LTQ Orbitrap XL mass spectrometry or immunoblotting.

### Nuclear run-on assay

Sorted cells were harvested in buffer containing 150 mM KCl, 10 mM Tris-HCl, 4 mM MgOAc with pH 7.4, followed by centrifugation to collect cell pellets. Pellets were lysed in buffer containing 150 mM KCl, 10 mM Tris-HCl, 4 mM MgOAc, and 0.5% NP-40, followed by sucrose density gradient centrifugation to prepare crude nuclei. Crude nuclei were incubated with 10 mM ATP, CTP, GTP, BrUTP, and RNase inhibitor at 28 °C for 5 min. RNAs were extracted using TRIzol reagent with manufacturer’s guidelines, followed by DNA digestion with DNaseI. RNA transcripts were immunoprecipitated with antibody against BrdU, followed by reverse transcription and RT-PCR analysis. 5 × 10^4^ cells were used for each single sample.

### Microarray assay

For circRNA microarray, ILC3s (Lin^−^CD90^high^CD45^low^) were isolated from *Rag1*^−/−^ mice treated with or without DSS. Then RNAs were isolated using Trizol reagent (Invitrogen) and used for Mouse circular RNA Array V2.0 assay (GSE142106). For mRNA microarray, RNAs from *circKcnt2*^+/^ and *circKcnt2*^−*/*−^ ILC3s were isolated using Trizol reagent (Invitrogen) and prepared for Affymetrix mRNA microarray assay (GSE142766) by Beijing Cnkingbio Biotechnology.

### RNA hybridization

Northern blotting was performed as previously described^4^. Briefly, after extracted using TRIzol, total RNA was subjected to formaldehyde-denaturing agarose electrophoresis, followed by transfer to positively charged nitrocellulose (NC) membrane with 20× SSC buffer (3.0 M NaCl and 0.3 M sodium citrate, pH 7.0). Then, the membrane was UV cross-linked and incubated with hybrid buffer for 2 h prehybridization, and then with biotin-labeled RNA probes (targeting circKcnt2: nt 262-127). Biotin signals were detected with HRP-conjugated streptavidin according to the manufacturer’s instructions (Thermo Scientific). For dot blotting, RNA was dropped onto Hybond-N^+^ membrane (GE Healthcare), followed by UV cross-linking. Then RNA signal was detected using biotin-labeled single-stranded DNA segment. RNA was generated by in vitro transcription.

### Fluorescence in situ hybridization

ILC3s were hybridized with fluorescence-conjugated *circKcnt2* probes (5′-ACCCAAAATATGTGAGACCAAGTGAAGATAAGGCA-3′) and then immune-stained with appropriate antibodies. Images were obtained with an Olympus FV1000 laser scanning confocal microscope.

### DNaseI accessibility assay

Nuclei were purified from ILC3s according to the manufacturer’s protocol with a nuclei-isolation kit (Sigma-Aldrich). Then nuclei were resuspended with DNaseI digestion buffer and treated with DNaseI (Sigma) at 37 °C for 5 min. Then 2× DNaseI stop buffer (20 mM Tris, pH 8.0, 4 mM EDTA, 2 mM EGTA) was added to stop reactions. DNA was extracted and measured by qPCR.

### Electrophoretic mobility shift assay

Biotin-labeled linearized *circKcnt2* were synthesized by T7 transcription in vitro. Probes and Mbd3 proteins were incubated in binding buffer and mobility shift assay was performed using a Light Shift Chemiluminescent RNA EMSA Kit (Thermo Scientific) according to the manufacturer’s protocol.

### ELISA assay

ILC3s were isolated and cultured for 24 h with indicated cytokines. Then supernatants were collected and cytokines were detected using ELISA kit (eBioscience) according to the manufacturer’s instructions.

### Statistical analysis

Data were analyzed by GraphPad Prism 6.0. All flow cytometry data were analyzed with FlowJo 7. Adobe Photoshop CC 14.0 and ImageJ 1.48 were used for figure presentation. For statistical evaluation, an unpaired Student’s *t*-test was applied for calculating statistical probabilities in this study. For all panels, at least three independent experiments were performed with similar results, and representative experiments are shown. Data were presented as mean ± SD. *P* values ≤0.05 were considered significant (*P < 0.05; **P < 0.01; ***P < 0.001); *P* > 0.05, non-significant (NS).

### Reporting summary

Further information on research design is available in the Nature Research Reporting Summary linked to this article.

## Supplementary information

Supplementary Information

Reporting Summary

## Data Availability

The circRNA and mRNA microarray data were deposited to the GEO database (GSE142106 and GSE142766). All other data are included in the supplementary information or available from the authors upon reasonable requests. Source data are provided with this paper.

## Source data

Source Data
