# Supplementary material for: An inducible circular RNA circKcnt2 inhibits ILC3 activation to facilitate colitis resolution
Source: Nat Commun. 2020 Aug 14;11:4076. doi: 10.1038/s41467-020-17944-5 (PMC7427797; doi:10.1038/s41467-020-17944-5)
Supplement: Supplementary file 1 — Supplementary Information [file 41467_2020_17944_MOESM1_ESM.pdf]

An inducible circular RNA *circKcnt2* inhibits ILC3 activation to facilitate colitis resolution

Liu *et al*.

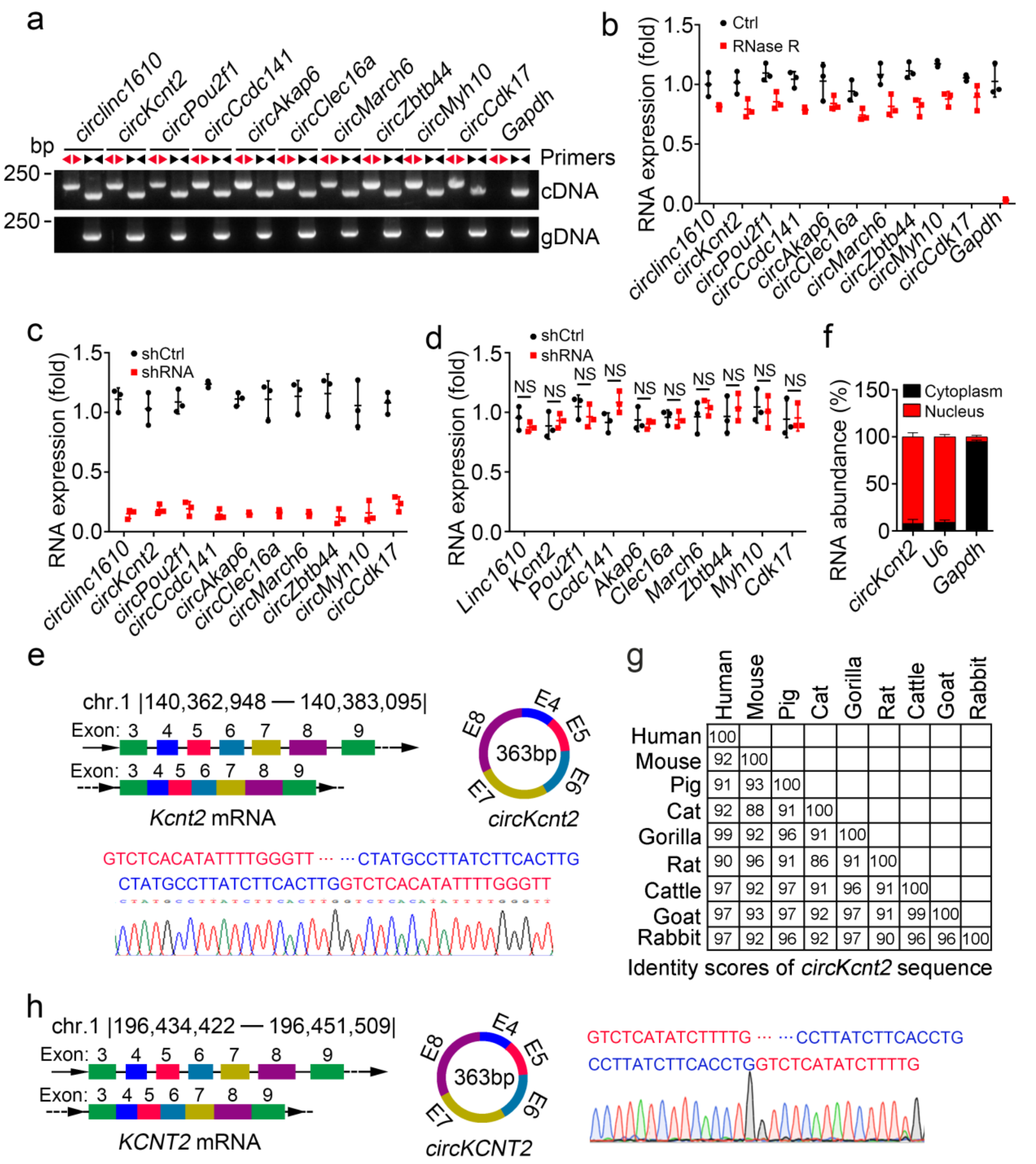

Supplementary Fig. 1. Validation of upregulated circRNAs in ILC3s. (a) Complementary DNA (cDNA) and genomic DNA (gDNA) were used as templates to amplify circRNAs in ILC3s using divergent and convergent primers. Red arrowheads, divergent primers; black arrowheads, convergent primers. (b) Total RNAs from ILC3s were treated with or without 3 U/μg RNase R for 1 h, followed by RNA extraction and RT-PCR analysis. n = 3 independent samples. (c) 14 days after DSS treatment, GFP^{+} ILC3s were isolated from *Rag1*$^{-/-}$*Il2rg*$^{-/-}$ mice for validation of knockdown efficiency via qRT-PCR. n = 3 independent samples. (d) Relative expression levels of indicated cognate linear transcripts against respective circRNAs were measured by qRT-PCR. NS, no significance. n = 3 independent samples. (e) Validation of *circKcnt2* in mouse ILC3s by DNA sequencing. Back-splicing site of *circKcnt2* was presented. n = 3 independent samples. (f) ILC3s isolated from DSS-treated *Rag1*$^{-/-}$ mice were subjected to nuclear and cytoplasmic separation, followed by RNA extraction and qRT-PCR analysis of *circKcnt2*.

n = 3 independent samples. (g) Sequence conservation of *circKcnt2* in indicated vertebrates. (h) Validation of *circKCNT2* in human LPL cells by DNA sequencing. Data were analyzed by an unpaired Student's *t*-test and shown as means ± SD. Data are representative of at least three independent experiments. Source data are provided as a Source Data file.

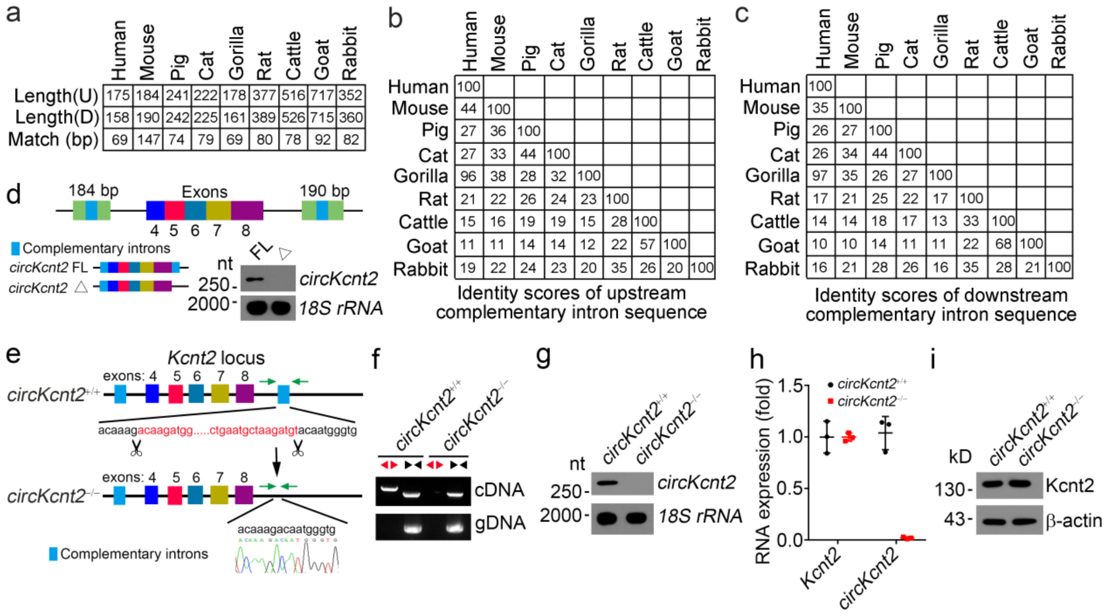

Supplementary Fig. 2. Construction of *circkcnt2*-deficient mice. (a) Information of complementary introns of *circKcnt2* in indicated vertebrates. U, upstream; D, downstream. (b, c) Analysis of conservation of upstream and downstream complementary intron sequences by DNAMAN software in indicated vertebrates. (d) Diagram of exons (exon4 to exon8) and complementary introns for *circKcnt2* formation. These exons and indicated complementary introns were cloned into pcDNA3 vector, followed by transfection into 293T cells for minigene assay. *circKcnt2* expression was examined by Northern blotting. 18S rRNA was used as a loading control. Minigene assay validated the requirement of intronic sequences for *circKcnt2* formation. (e) Construction diagram of *circKcnt2* knockout mice. (f-h) *CircKcnt2* deletion was confirmed by PCR, Northern blotting and qRT-PCR in ILC3s. Red arrowheads denote primers for *circKcnt2* detection. Black arrowheads denote primers for liner Kcnt2 detection. n = 3 independent samples. (i) Kcnt2 expression in ILC3s was tested by Western blotting. Data were analyzed by an unpaired Student's *t*-test and shown as means ± SD. Data are representative of at least three independent experiments. Source data are provided as a Source Data file.

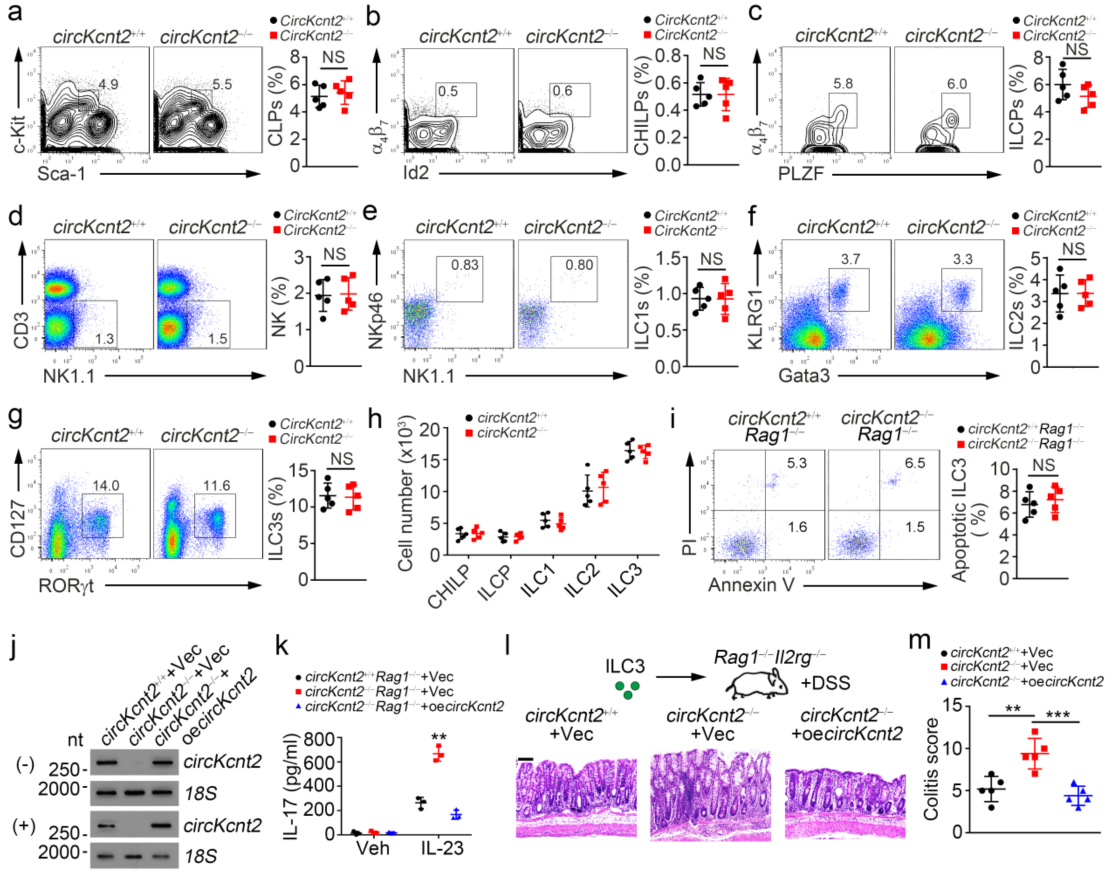

Supplementary Fig. 3. *circKcnt2* deletion does not affect ILC3 development and apoptosis. (a-c) Analyses of CLPs, CHILPs and ILCPs in *circKcnt2*$^{+/+}$ and *circKcnt2*$^{-/-}$ mice. CLP was gated on Lin^{-}CD127^{+}Sca-1^{low}c-Kit^{low}Flt3^{+}. CHILP was gated on Lin^{-}CD127^{+}Flt3^{-}CD25^{-}Id2$^{+}\alpha_4\beta_7^{+}$. ILCP was gated on Lin^{-}Flt3^{-}CD127^{+}c-Kit$^{+}\alpha_4\beta_7^{+}$PLZF^{+}. n = 5 independent samples. (d-g) FACS analyses of NK, ILC1, ILC2 and ILC3 in the small intestines of *circKcnt2*$^{+/+}$ and *circKcnt2*$^{-/-}$ mice. NK was gated on CD127^{-}CD19^{-}CD3^{-}NK1.1^{+}. ILC1 was gated on CD3^{-}CD19^{-}CD127^{+}NK1.1^{+}NKp46^{+}. ILC2 was gated on Lin^{-}CD127^{+}CD90^{+}KLRG1^{+}Gata3^{+}. ILC3 was gated on Lin^{-}RORγt^{+}CD45^{low}CD127^{+}. n = 5 independent samples. (h) Numbers of indicated cell types in *circKcnt2*$^{+/+}$ and *circKcnt2*$^{-/-}$ mice. n = 5 independent samples. (i) Detection of ILC3 apoptosis in *circKcnt2*$^{+/+}$*Rag1*$^{-/-}$ and *circKcnt2*$^{-/-}$*Rag1*$^{-/-}$ mice after DSS treatment. n = 5 independent samples. (j) ILC3s isolated from *circKcnt2*$^{+/+}$ and *circKcnt2*$^{-/-}$ mice were infected with *circKcnt2*-overexpressing lentivirus (pSIN-EF2-GFP) or control lentivirus. *circKcnt2* expression in infected ILC3s with or without RNase R treatment was detected by Northern blotting using back-splicing junction-specific biotin-labeled probe (targeting circKcnt2: nt 262 ~ 127). (-): without RNase R; (+) with RNase R. (k) Indicated ILC3s were infected with *circKcnt2*-overexpressing lentivirus (pSIN-EF2-GFP) or control. ELSIA assay was performed at the presence of IL-23 or Vehicle (Veh) (*** P = 0.0008). Vec, Vector. n = 3 independent samples. (l) H&E staining of colon tissues. *CircKcnt2*$^{-/-}$ ILC3s were isolated and infected with lentivirus for circKcnt2 restoration. ILC3s were transplanted into *Rag1*$^{-/-}$*Il2rg*$^{-/-}$ mice, followed by 3% DSS treatment. Scale bar, 100 μm. (m) Colitis scores of indicated mice as in K. n=5 for each

group (** $P = 0.0039$; *** $P = 0.0008$). ** $P<0.01$. Data were analyzed by an unpaired Student's *t*-test and shown as means ± SD. Data are representative of at least three independent experiments. Source data are provided as a Source Data file.

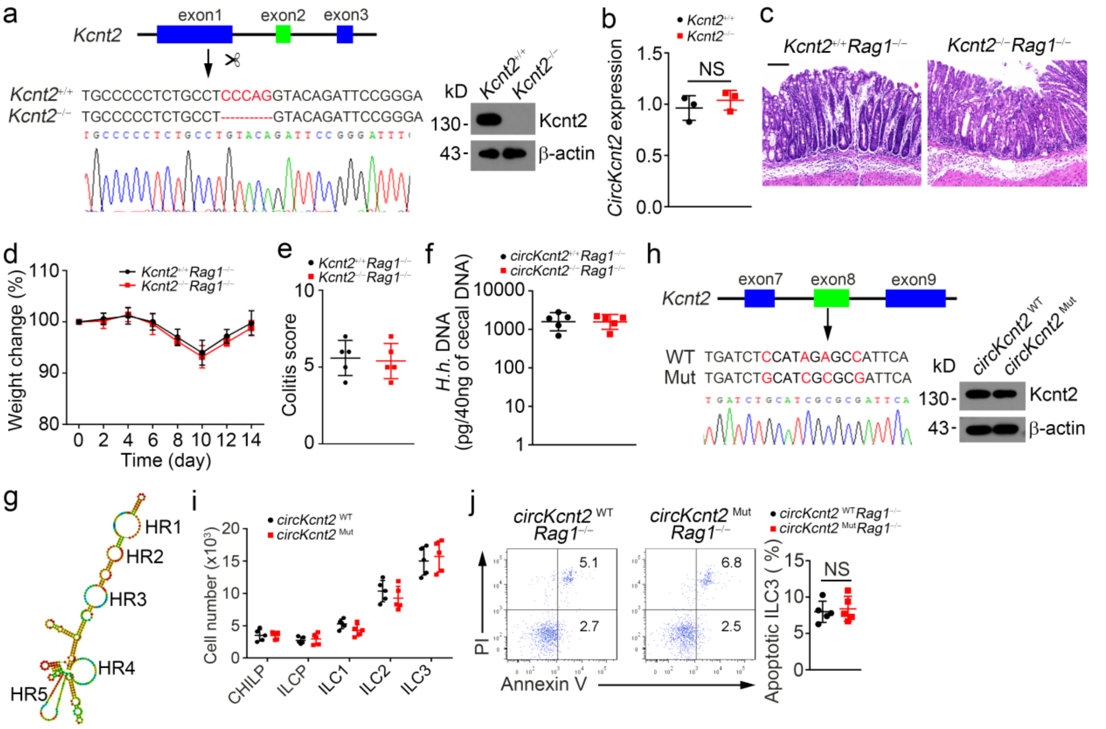

Supplementary Fig. 4. Analysis of Kcnt2 deletion during ILC3-mediated inflammation. (a) Validation of Kcnt2 deletion in ILC3s isolated from *Kcnt2*$^{-/-}$ mice by Western blotting. (b) qRT-PCR analysis for *circKcnt2* expression in *Kcnt2*$^{+/+}$ and *Kcnt2*$^{-/-}$ ILC3s (P = 0.4355). (c) *Kcnt2*$^{+/+}$*Rag1*$^{-/-}$ and *Kcnt2*$^{-/-}$*Rag1*$^{-/-}$ mice were treated with 3% DSS, and colons were isolated and analyzed by H&E staining. Scale bar, 100 μm. (d) Body weight changes of DSS-treated *Kcnt2*$^{+/+}$*Rag1*$^{-/-}$ and *Kcnt2*$^{-/-}$*Rag1*$^{-/-}$ mice were calculated. n=5 for each group. (e) Colitis scores of indicated mice treated as in (c). n=5 for each group. (f) Analysis of *H.h.* DNA in cecal contents of infected *circKcnt2*$^{+/+}$*Rag1*$^{-/-}$ and *circKcnt2*$^{-/-}$*Rag1*$^{-/-}$ mice by qRT-PCR. n=5 for each group. (g) Prediction of stem-loop structures of *circKcnt2* by using RNAfold WebServer (http://rna.tbi.univie.ac.at/). Predictions were based on minimum free energy (MFE) and partition function. HR, hairpin region. (h) Construction strategy for *circKcnt2*-mutant (*circKcnt2*^{Mut}) mice. Western blotting analysis of Kcnt2 in *circKcnt2*^{WT} and *circKcnt2*^{Mut} ILC3s. (i) Analyses of CHILPs, ILCPs, ILC1s, ILC2s and ILC3s in *circKcnt2*^{WT} and *circKcnt2*^{Mut} mice. n = 5 independent samples. (j) Apoptosis of *circKcnt2*^{WT}*Rag1*$^{-/-}$ and *circKcnt2*^{Mut}*Rag1*$^{-/-}$ ILC3s were analyzed by Annexin V/PI staining after 3% DSS treatment (P = 0.7028). n = 5 independent samples. Data Data were analyzed by an unpaired Student's t-test and shown as means ± SD. Data are representative of at least three independent experiments. Source data are provided as a Source Data file.

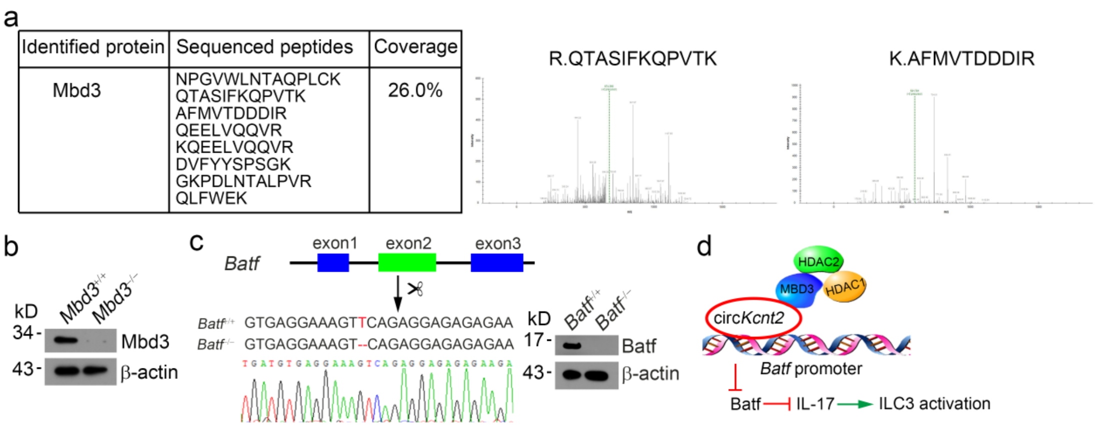

Supplementary Fig. 5. Mbd3 deletion promotes ILC3-mediated innate colitis. (a) Peptides of Mbd3 were identified by mass spectrometry. (b) Western blotting analysis of Mbd3 expression in *Mbd3*$^{+/+}$ and *Mbd3*$^{-/-}$ ILC3s. (c) Construction strategy of *Batf*-deficient mice. (d) Work model of *circKcnt2*-mediated resolution of innate colitis. *CircKcnt2* recruits the NURD complex onto *Batf* promoter to inhibit its expression, which suppresses *Il17* expression for ILC3 inactivation to promote innate colitis resolution. Data Data were analyzed by an unpaired Student's *t*-test and shown as means ± SD. Data are representative of at least three independent experiments.

Supplementary Table 1. Mutation sequences for *circKcnt2* loops.

Loop	Wild-type sequence	Mutant sequence
HR1	5’-GTCTCACAT-3’	5’-GTAACCCCA-3’
HR2	5’-CTCAGTG-3’	5’-GAAGGTG-3’
HR3	5’-ATTTTGATATCT-3’	5’-TTTCAAATAGCT-3’
HR4	5’-TTAATGATCT-3’	5’-ATGGCTCTAT-3’
HR5	5’-TCTCAATATTCTGGCCC-3’	5’-ACTCATTATTCTAATAG-3’

Supplementary Table 2. sgRNAs for CRISPR/Cas9-mediated mouse construction

Genes	sgRNA sequences
circKcnt2 (up)	5'-ATTGTCATACAAAGACAAGATGG-3'
circKcnt2 (down)	5'-CTGAATGCTAAGATGTACAATGG-3'
Batf	5'-ATGATGTGAGGAAAGTTCAGAGG-3'
Kcnt2	5'-TCCCGGAATCTGTACCTGGGAGG-3'
circKcnt2^{Mut}	5'-TGCAGACTGTGTACGCTGAATGG-3'

Supplementary Table 3. Primers for PCR and qRT-PCR. (Co: Convergent primer; Di: Divergent primer)

Genes	Forward	Reverse
circKcnt2 (Di)	5'-TTAATGATCTCCATAGAGCC-3'	5'-CCTGTAAACCCCATAAAGGT-3'
circKcnt2 (Co)	5'-TATAAGGGAAATATCTGGGA-3'	5'-CTTAAGGTGGGCCAGAATAT-3'
circlinc1610(Di)	5'-ATTGATGAAAATCACCTCAG-3'	5'-TGTGGCCCACACCCATAATC-3'
circlinc1610(Co)	5'-TGTATCTTCTAATCCACCAA-3'	5'-AGGTGCTTCAAGCGGCATGT-3'
circPou2f1(Di)	5'-ACAGACAGACAAAACTAAGT-3'	5'-TGCAAAAGATGGATAAGAGA-3'
circPou2f1(Co)	5'-TAATCAGAATAGTAGATGTG-3'	5'-TGAAAGGATATACTAGACAAG-3'
circCcdc141(Di)	5'-TCCTCGTGCTTGCAGATTAG-3	5'-GGGGTAAAGCCCATTCCGTT-3'
circCcdc141(Co)	5'-AGGACCTGATAGTCAAAGCA-3'	5'-TCCTTTCCTGCCAAGTCCTT-3'
circAkap6(Di)	5'-GAAGATCGACATAGTAACAT-3'	5'-TCTGTCTCATCCCATTCCAG-3'
circAkap6(Co)	5'-TGGTTGACATCCTATAAGAG-3'	5'-TCCTCTTGCTCAGCTCACCA-3'
circClec16a(Di)	5'-ACTGACTCGGGAGGAGGACCT-3'	5'-GCGCTCTTCTCCTCGTCTGT-3'
circClec16a(Co)	5'-GTGCCAAGTGAAGCTGAGAA-3'	5'-ACCGGATCCTGCCATCTGGC-3'
circMarch6(Di)	5'-TATTGATGGTCGTTGTGCTG-3'	5'-CAGTACATACACAAGGATGA-3'
circMarch6(Co)	5'-TAGGAGGAAATGGTGCAGAA-3'	5'-TTGACCGTCCTGGGCATCAG-3'
circZbtb44(Di)	5'-TCACTGAAGGATTGCCTACA-3'	5'-AGGAGCTGTGAGTAAATGTT-3'
circZbtb44(Co)	5'-GCTAGTACATGCTCAGAGTT-3'	5'-TGGAGCTATTTTCTTGTCCA-3'
circMyh10(Di)	5'-TCGTCAAGCTAAAGATGAGC-3'	5'-TGTGAGAAGAAGCAACGTGG-3'
circMyh10(Co)	5'-CAATTCTGGAATCCTTTGGA-3'	5'-CAATATAGCCAGTTACATCA-3'
circCdk17(Di)	5'-AGAGGAGACTTAAAACTTGG-3'	5'-TGCAGTTCATCTTCCACAGT-3'
circCdk17(Co)	5'-CCACAACCTCTAATTAATCA-3'	5'-CTGCCGGAACCCTCTTCTTA-3'
Il22	5'-CAACTGTTGACACTTGTGCGA-3'	5'-TGACGATGTATGGCTGCTGG-3'
Il17a	5'-GCTGACCCCTAAGAAACCCC-3'	5'-GAAGCAGTTTGGGACCCCTT-3'
Ifng	5'- AGACAATCAGGCCATCAGCA-3'	5'-TGGACCTGTGGGTTGTTGAC-3'
Batf	5'-TGGGAGAGCCCGGAAGATTA-3'	5'-ATGAGTCCTGTTTGCCAGGG-3'
Mid1	5'-TGATTGAGCCAATCCCGGAC-3'	5'-CTGGTCATCGGTCACACAGT-3'
Zfp189	5'-GAAGGCTCCGGGAACTTGAC-3'	5'-TCGCCTCGTTCAGGAATAGC-3'
Blzf1	5'-TAAGCGTCCATACCGCTTGC-3'	5'-CGCCTCGCTCACTCTAATCA-3'
Tcf19	5'-TCCTTGGCTGTCAAACCTCG-3'	5'-GTGTGACACGGGTCTTCTCC-3'
Ankra2	5'-CAGCTGATCGTGGAAGAGTGT-3'	5'-TTTGGGTCTAGCTGGTGCTC-3'
E2f3	5'-TCGGAAATGCCCTTACAGCA-3'	5'-ACTTCTTGGTGAGCAGACCG-3'
Clock	5'-AACTCCCAGTCAGTTGGTCC-3'	5'-ATGCTGCATGGCTCCTAACT-3'
Zfp235	5'-GAAATGTTCCAGCGCCAGTG-3'	5'-CCCGAGAAGACACACCGAAA-3'
Zfp27	5'-TCATCCGAGATGATTCCGCA-3'	5'-GCATGATCACGCTGGGTCTA-3'
Kcnt2	5'-TGCTGCATGCTAATTGCTTTGT-3'	5'-AGCATTTTCCACATCCATGACT-3'
Gaphd(Di)	5'-GCTGAGTATGTCGTGGAGTCT-3'	5'-GGCAGCCCTGGTGACCAGGCGC-3'
Gaphd(Co)	5'-GCGCCTGGTCACCAGGGCTGCC-3'	5'-AGACTCCACGACATACTCAGC-3'
18S	5'-AACCCGTTGAACCCCATT-3'	5'-CCATCCAATCGGTAGTAGCG-3'

Supplementary Table 4. Primers for ChIP analysis

Genes	Forward	Reverse
Batf pro#1	5’-GCTCAGGGGACGCTTGATTA-3’	5’-AGAGCACTTATCGTAGCCGC-3’
Batf pro#2	5’-ATCCACGTACCGAACTTATC-3’	5’-CCAGGCACACACCTGAGGTG-3’
Batf pro#3	5’-CACTTGACACTCCCTGATGT-3’	5’-AATAAAAGTCAATCCTTGTA-3’
Batf pro#4	5’-CAGGCAGAATTGTAATCAGT-3’	5’-TCCCAGTGTGGCTCAAATGC-3’
Batf pro#5	5’-GTACCCGAGGAGTCAGGAAG-3’	5’-GCTGCTCGTCCAGAGGACTT-3’
Batf pro#6	5’-CGTGTTCGTGTCATGGGACA-3’	5’-CTGCAATGAGGGATGACAAT-3’
Batf pro#7	5’-GATCTGACTCCAAGTCCAGA-3’	5’-TCTTCAGAAGTCAGTGACGT-3’
Batf pro#8	5’-TGCTTGGTCATCTGTAAAAG-3’	5’-ATGACCGGCCTTCCTCCTTC-3’
Batf pro#9	5’-AGGCCTGTGACCTGGAGGCT-3’	5’-GGCCAGAATTAGCAAGTTCT-3’
Batf pro#10	5’-CTTCATCAATTGAAGAGCGC-3’	5’-ACCACAGAATTAGGTGAAAG-3’

Supplementary Table 5. shRNA sequences used in this study

Genes	shRNA sequences
circKcnt2	5'-TATCTTCACTTGGTCTCACAT-3'
circlinc1610	5'-TGGAATTCTCTGGAACTTTGT-3'
circPou2f1	5'-GCTAAGACTCAAGAATGAATA-3'
circCcdc141	5'-GTGCTAACAAGGTTTCTTCTT-3'
circAkap6	5'-CATTGAAAGGAGACTCTGAAT-3'
circClec16a	5'-GATGTCTTGGATCTGAAGATT-3'
circMarch6	5'-CACATTAAAAATGACAAATAT-3'
circZbtb44	5'-AGATGACGATGACAGGATAAT-3'
circMyh10	5'-GGAACACCTGAAATGGGTGAA-3'
circCdk17	5'-CAAGAATTCTGAAGGGGAGTT-3'
