# Supplementary material for: An inducible circular RNA circKcnt2 inhibits ILC3 activation to facilitate colitis resolution
Source: Nat Commun. 2020 Aug 14;11:4076. doi: 10.1038/s41467-020-17944-5 (PMC7427797; doi:10.1038/s41467-020-17944-5)
Supplement: Supplementary file 2 — Reporting Summary [file 41467_2020_17944_MOESM2_ESM.pdf]

nature research

Corresponding author(s): Zusen Fan

Last updated by author(s): Jul 27, 2020

Reporting Summary

Nature Research wishes to improve the reproducibility of the work that we publish. This form provides structure for consistency and transparency in reporting. For further information on Nature Research policies, seeAuthors & Referees and theEditorial Policy Checklist .

Statistics

For all statistical analyses, confirm that the following items are present in the figure legend, table legend, main text, or Methods section.

n/a	Confirmed	
☐	☒	The exact sample size (*n*) for each experimental group/condition, given as a discrete number and unit of measurement
☐	☒	A statement on whether measurements were taken from distinct samples or whether the same sample was measured repeatedly
☐	☒	The statistical test(s) used AND whether they are one- or two-sided<br>*Only common tests should be described solely by name; describe more complex techniques in the Methods section.*
☐	☒	A description of all covariates tested
☒	☐	A description of any assumptions or corrections, such as tests of normality and adjustment for multiple comparisons
☐	☒	A full description of the statistical parameters including central tendency (e.g. means) or other basic estimates (e.g. regression coefficient) AND variation (e.g. standard deviation) or associated estimates of uncertainty (e.g. confidence intervals)
☐	☒	For null hypothesis testing, the test statistic (e.g. *F*, *t*, *r*) with confidence intervals, effect sizes, degrees of freedom and *P* value noted<br>*Give P values as exact values whenever suitable.*
☒	☐	For Bayesian analysis, information on the choice of priors and Markov chain Monte Carlo settings
☐	☒	For hierarchical and complex designs, identification of the appropriate level for tests and full reporting of outcomes
☒	☐	Estimates of effect sizes (e.g. Cohen's *d*, Pearson's *r*), indicating how they were calculated

Our web collection on statistics for biologists contains articles on many of the points above.

Software and code

Policy information about availability of computer code

Data collection	No software was used.
Data analysis	Data were analyzed by GraphPad Prism 6.0. All flow cytometry data were analyzed with FlowJo 7. Adobe Photoshop CC 14.0 and ImageJ 1.48 were used for Figure presentation. One-tailed unpaired Student's t-test was performed using GraphPad Prism 6.0. No computer code was used.

For manuscripts utilizing custom algorithms or software that are central to the research but not yet described in published literature, software must be made available to editors/reviewers. We strongly encourage code deposition in a community repository (e.g. GitHub). See the Nature Research guidelines for submitting code & software for further information.

Data

Policy information about availability of data

All manuscripts must include a data availability statement. This statement should provide the following information, where applicable:

- Accession codes, unique identifiers, or web links for publicly available datasets
- A list of figures that have associated raw data
- A description of any restrictions on data availability

The circRNA and mRNA microarray data were deposited to the GEO database (GSE142106 and GSE142766).

Field-specific reporting

Please select the one below that is the best fit for your research. If you are not sure, read the appropriate sections before making your selection.

Life sciences study design

All studies must disclose on these points even when the disclosure is negative.

Sample size	Where statistical analysis was applied, independent triplicate experiments were carried out.
Data exclusions	No data were excluded from the anaylses.
Replication	All experiments were prepared with biological triplicate and all attempts at replication were successful.
Randomization	For imaging experiments, each cell was randomly chosen; for other experiments randomization is not relevant as cells in bulk were used for western blot, qPCR or flow cytometry etc.
Blinding	There is no special blinding as the analyses were performed by machine or software where blinding is not necessary to reduce bias.

Reporting for specific materials, systems and methods

We require information from authors about some types of materials, experimental systems and methods used in many studies. Here, indicate whether each material, system or method listed is relevant to your study. If you are not sure if a list item applies to your research, read the appropriate section before selecting a response.

Materials & experimental systems

n/a	Involved in the study	
☐	☒	Antibodies
☐	☒	Eukaryotic cell lines
☒	☐	Palaeontology
☐	☒	Animals and other organisms
☒	☐	Human research participants
☒	☐	Clinical data

Methods

n/a	Involved in the study	
☒	☐	ChIP-seq
☐	☒	Flow cytometry
☒	☐	MRI-based neuroimaging

Antibodies

Antibodies used	Anti-H3K27ac (Cat# 8173), anti-Batf (Cat# 8638), anti-H3K27me3 (Cat# 9733), anti-Mbd3 (Cat# 99169), anti-Chd3 (Cat# 4241), anti-Rbap46 (Cat# 6882), anti-Hdac1 (Cat# 34589), anti-Hdac2 (Cat# 57156), anti-EEA1 (Cat# 3288), anti-H3 (Cat# 4499) and anti-β-actin (Cat# 3700) were from Cell Signaling Technology (Danvers, USA). Anti-Kcnt2 (Cat# bs-12177R) was from Bioss Antibodies (Beijing, China). Anti-CD127 (A7R34), anti-c-Kit (2B8), anti-CD3 (17A2), anti-CD4 (GK1.5), anti-CD19 (1D3), anti-NK1.1 (PK136), anti-CD150 (mShad150), anti-CD34 (RAM34), anti-CD45 (30-F11), anti-CD90 (HIS51), anti-Sca-1 (D7), anti-CD25 (PC61.5), anti-Flt3 (A2F10), anti-α4β7 (DATK32), anti-RORγt (AFKJS-9), anti-NKp46 (29A1.4), anti-Gata3 (TWAJ), anti-KLRG1 (2F1), anti-PLZF (Mags.21F7), Lineage cocktail (88-7772-72), anti-CD48 (HM48-1), Anti-IL-17 (eBio17B7), and anti-CD16/32 (93) were purchased from eBiosciences (San Diego, USA). Anti-BrdU (600-401-C29) was purchased from ThermoFisher.
Validation	Antibodies were previously validated as per the manufacturer's website or published papers cited in this manuscript.

Eukaryotic cell lines

Policy information about cell lines

Cell line source(s)	HEK293T cell line was obtained from ATCC.
Authentication	None of the cell lines were authenicated.
Mycoplasma contamination	HEK293T cells were not tested for mycroplasma.
Commonly misidentified lines (See ICLAC register)	none used

Animals and other organisms

Policy information about studies involving animals; ARRIVE guidelines recommended for reporting animal research

Laboratory animals	circKcnt2-/-, Batf-/-, Mbd3flox/flox, PLZF-GFPcre, Rorc(γt)+/GFP and Id2+/GFP mice were used in this study. Sex-matched littermates were used for all experiments. Animals were housed under pathogen-free conditions. Experiments were performed in accordance with protocols approved by the Institutional Committee of Institute of Biophysics, Chinese Academy of Sciences.
Wild animals	No wild animals were used in this study.
Field-collected samples	This study did not involve sample collected from the field.
Ethics oversight	All experiments involving mice were approved by the institutional committee of Institute of Biophysics, Chinese Academy of Sciences.

Note that full information on the approval of the study protocol must also be provided in the manuscript.

Flow Cytometry

Plots

Confirm that:

- [x] The axis labels state the marker and fluorochrome used (e.g. CD4-FITC).
- [x] The axis scales are clearly visible. Include numbers along axes only for bottom left plot of group (a 'group' is an analysis of identical markers).
- [x] All plots are contour plots with outliers or pseudocolor plots.
- [x] A numerical value for number of cells or percentage (with statistics) is provided.

Methodology

Sample preparation	The peyers patches (PPs) were removed from the small intestine. Then the intestine was cut open longitudinally, followed by wash using phosphate buffer saline (PBS) five times. Afterwards, the intestine was cut into pieces and washed using solution I buffer (10 mM HEPES and 5mM EDTA in HBSS) five times, followed by digestion using solution II buffer (DNaseI, 5% FBS, 0.5 mg/ml collagenase II and collagenase III) three times. Finally, LPLs were sifted through 70m strainers and utilized for FACS analyses
Instrument	FACSAria III instrument (BD Biosciences)
Software	Flowjo 7.6.1 software
Cell population abundance	1~2x10^5 cells were recorded by the cytometer for each sample.
Gating strategy	For flow cytometric analysis, CLP (Lin-CD127+Sca-1lowc-KitlowFlt3+), CHILP (Lin-CD127+Flt3-CD25-Id2GFPα4β7+), ILCP (Lin-Flt3-CD127+c-Kit+α4β7+PLZF+), ILC1 (CD3-CD19-CD127+NK1.1+NKp46+), ILC2 (Lin-CD127+CD90+KLRG1+Gata3+), ILC3 (Lin-RORγt+CD127+ or Lin-CD90highCD45low) populations were sorted with a FACSAria III instrument (BD Biosciences).

- [x] Tick this box to confirm that a figure exemplifying the gating strategy is provided in the Supplementary Information.
